# The accessory protein TagV is required for full Type VI secretion system activity in *Serratia marcescens*

**DOI:** 10.1111/mmi.15027

**Published:** 2023-01-23

**Authors:** Mark Reglinski, Laura Monlezun, Sarah J. Coulthurst

**Affiliations:** 1 Division of Molecular Microbiology, School of Life Sciences, University of Dundee, Dundee, United Kingdom DD1 5EH

**Keywords:** Type VI secretion system, bacterial protein secretion, Type VI accessory genes, *Serratia marcescens*

## Abstract

The bacterial Type VI secretion system (T6SS) is a dynamic macromolecular structure that promotes inter- and intra-species competition through the delivery of toxic effector proteins into neighbouring cells. The T6SS contains fourteen well-characterised core proteins necessary for effector delivery (TssA-M, PAAR). In this study we have identified a novel accessory component required for optimal T6SS activity in the opportunistic pathogen *Serratia marcescens*, which we name TagV. Deletion of *tagV*, which encodes an outer membrane lipoprotein, caused a reduction in the T6SS-dependent antibacterial activity of *S. marcescens* Db10. Mutants of *S. marcescens* lacking the core component TssJ, a distinct outer membrane lipoprotein previously considered essential for T6SS firing, retained a modest T6SS activity that could be abolished through deletion of *tagV*. TagV did not interact with the T6SS membrane complex proteins TssL or TssM, but is proposed to bind to peptidoglycan, indicating that the mechanism by which TagV promotes T6SS firing differs from that of TssJ. Homologues of *tagV* were identified in several other bacterial genera, suggesting that the accessory function of TagV is not restricted to *S. marcescens*. Together, our findings support the existence of a second, TssJ-independent mechanism for T6SS firing that is dependent upon the activity of TagV proteins.

## Introduction

The Type VI secretion system (T6SS) is a contractile nanoweapon that plays an important role in bacterial inter- and intra-species competition (Coulthurst, 2019). The basic T6SS machinery comprises fourteen well-characterised core components (denoted TssA-M and PAAR), which form four distinct macromolecular structures: the baseplate, membrane complex (MC), contractile sheath and expelled puncturing device. According to the current model (Wang *et al*., 2019), T6SS assembly is initiated by the outer membrane lipoprotein TssJ, which stimulates formation of the MC through the recruitment of TssL and TssM (Rapisarda *et al*., 2019). The MC recruits the cytoplasmic baseplate (comprising TssE, TssF, TssG, TssK and the VgrG/PAAR spike) via interactions with TssK, and it is anchored to the cell wall through a peptidoglycan binding interaction that is mediated by TssL in most systems described to date (Nguyen *et al*., 2021, Wang *et al*., 2018, Rapisarda *et al*., 2019). The puncturing device comprises a tube of stacked hexameric Hcp rings topped with a VgrG/PAAR spike that facilitates penetration of the target cell envelope. The Hcp tube assembles onto the base of VgrG, surrounded by a helical sheath made up of TssB/TssC subunits, and extends away from the baseplate across the cytoplasm. Subsequently, the extended TssBC sheath contracts in a rapid and powerful step, known as T6SS “firing”, that drives the puncturing device, loaded with effector proteins, into a neighbouring target cell (Wang *et al*., 2019). The major structures underpinning T6SS firing share structural and functional homology with those involved in bacteriophage tail contraction, with the exception of the MC, which is required to anchor the T6SS in the cell envelope and allow the puncturing device to pass through both membranes without loss of their integrity and barrier function (Rapisarda *et al*., 2019, Durand *et al*., 2015). The MC comprises two concentric rings of TssM pillars which span the periplasmic space and are linked to TssJ (and thus the outer membrane) via the C-terminal domain (Durand *et al*., 2015). The hydrophobic N-terminal domain of TssM and the inner membrane protein TssL form a periplasmic “gate” that is assembled in a tightly closed state and undergoes a marked conformational change to facilitate trans-envelope channel formation upon T6SS firing (Rapisarda *et al*., 2019).

In addition to the highly conserved T6SS core components described above, many T6SSs contain “accessory proteins” (often called “Tag” proteins) that are essential for full T6SS function. These proteins are usually encoded within the main T6SS gene cluster and have been shown to play a variety of important roles, including post-translational regulation of system assembly (TagE-H; Mougous *et al*., 2007), sheath stabilisation (TagA and B; Santin *et al*., 2018) and detection of incoming attacks (TagQ-T; Basler *et al*., 2013). Several Tag proteins interact with peptidoglycan, including TagL which anchors the MC to the cell wall in a subset of strains encoding a truncated version of TssL, and TagX, a specialised endopeptidase that allows the T6SS machine to traverse the cell wall in some species (Weber *et al*., 2016, Santin *et al*., 2019). Several other putative peptidoglycan binding Tags have been described (for example TagI, TagM and TagW) whose functions are yet to be elucidated (Table S1).

T6SSs are present in approximately 25% of Gram-negative species and have been shown to deliver toxic “effector” proteins into bacterial, fungal, and eukaryotic cells in association with the puncturing device (Ho *et al*., 2014, Monjaras Feria & Valvano, 2020, Jurenas & Journet, 2021). Effectors interact non-covalently with Hcp, VgrG or PAAR proteins (cargo effectors) or may be covalently attached to these proteins (specialised effectors), and are situated inside the Hcp tube or on the outside of the VgrG/PAAR spike (Jurenas & Journet, 2021). Anti-bacterial effectors include a variety of enzymes that hydrolyse peptidoglycan, phospholipids, DNA, and cellular cofactors, whilst other effectors scavenge metal ions from the extracellular environment or act against eukaryotic cells, for example by cross-linking actin in host cells or inhibiting fungal competitors (Jurenas & Journet, 2021, Monjaras Feria & Valvano, 2020). Thus, the T6SS is a highly versatile system that can be armed with different sets of effectors in order to target competing microorganisms and/or mammalian host cells.

*Serratia marcescens* is a Gram-negative bacillus that can colonise a variety of environmental and animal niches and is also an opportunistic human pathogen representing a significant cause of hospital-acquired infections (Ono *et al*., 2022). Given the range of polymicrobial settings in which *S. marcescens* resides, it is unsurprising that it possesses an efficient T6SS that delivers multiple anti-microbial effectors with functions including peptidoglycan and DNA degradation and membrane depolarisation (Alcoforado Diniz & Coulthurst, 2015, English *et al*., 2012, Mariano *et al*., 2019, Trunk *et al*., 2018). Together these effectors should allow *S. marcescens* to compete for dominance against many different bacterial and fungal species across a range of niches (Hernandez *et al*., 2020, Murdoch *et al*., 2011, Trunk *et al*., 2018).

While the T6SS core components and effectors of the model *S. marcescens* strain Db10 are well characterised, the function of the protein encoded by the gene *SMDB11_2251*, located immediately upstream of *tssJ* in the main T6SS gene cluster, remains to be elucidated. Here we demonstrate that *SMDB11_2251* encodes a novel accessory lipoprotein that is required for optimal T6SS function in *S. marcescens* and is conserved in other T6SS encoding bacterial species. We have renamed this protein “TagV” in line with the nomenclature established previously (Shalom *et al*., 2007) and provide evidence that TagV mediates T6SS firing via a previously-undescribed TssJ-independent pathway.

## Results

### TagV is a conserved outer membrane lipoprotein that is essential for full T6SS activity in *S. marcescens*

**Figure 1:**
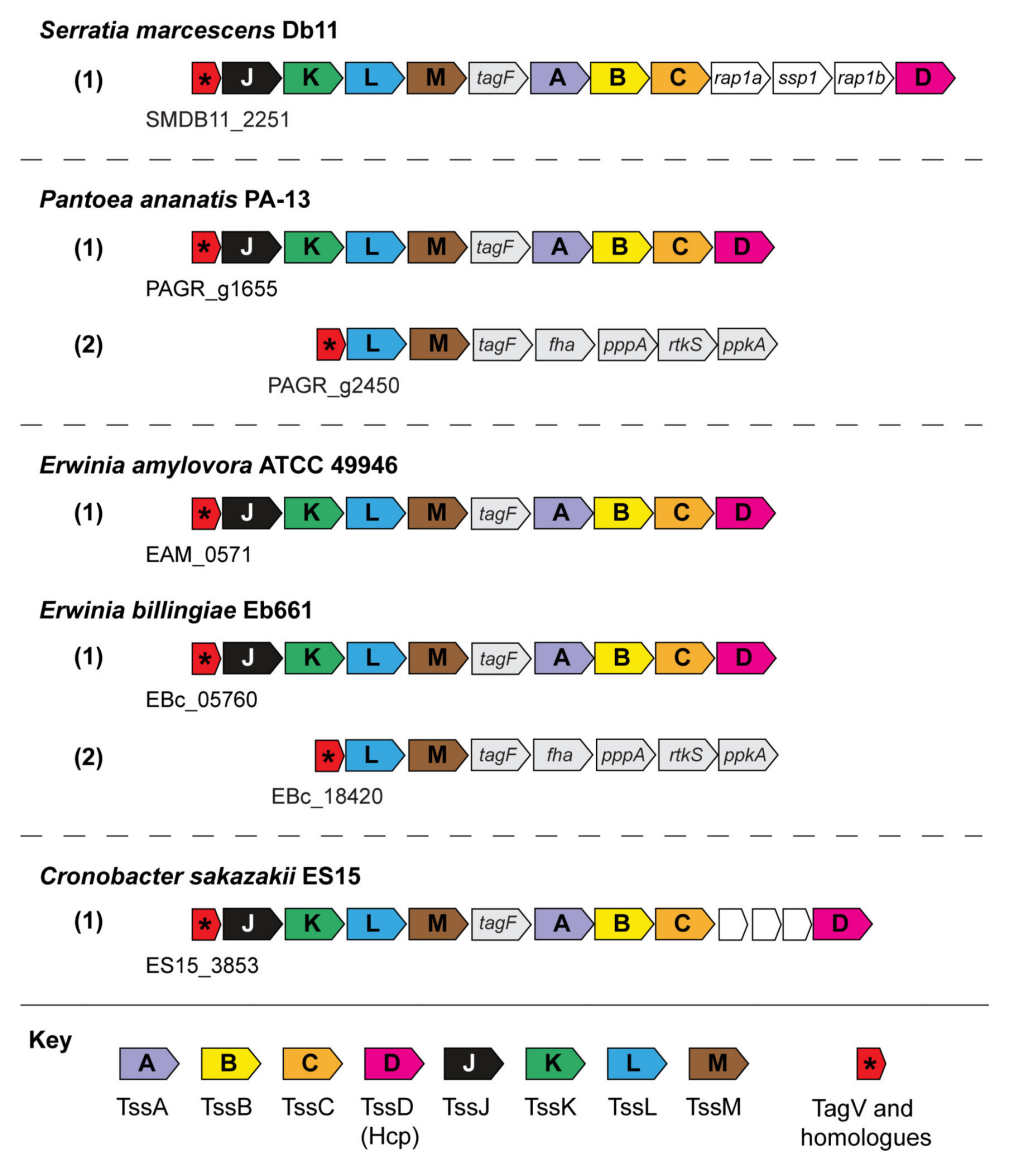
The genetic context of *tagV* homologues in several bacterial species. Genes encoding core T6SS components are coloured and labelled with their corresponding Tss designation. Genes encoding selected T6SS accessory components (*tagF, fha, pppA, ppkA* and *rtkS*) and effector/immunity pairs (*rap1a, ssp1* and *rap1b*) are also labelled. Genomic identifiers corresponding to the open reading frame encoding TagV from *S. marcescens* Db11 (accession: HG326223) and its homologues from *P. ananatis* PA13 (accession: AER32178), *E. amylovora* ATCC49946 (accession: CBJ45246) *E. billingiae* Eb661 (accession: CAX59373) and *C. sakazakii* ES15 (accession: AFK01427) are provided under each T6SS gene cluster.

**Figure 2:**
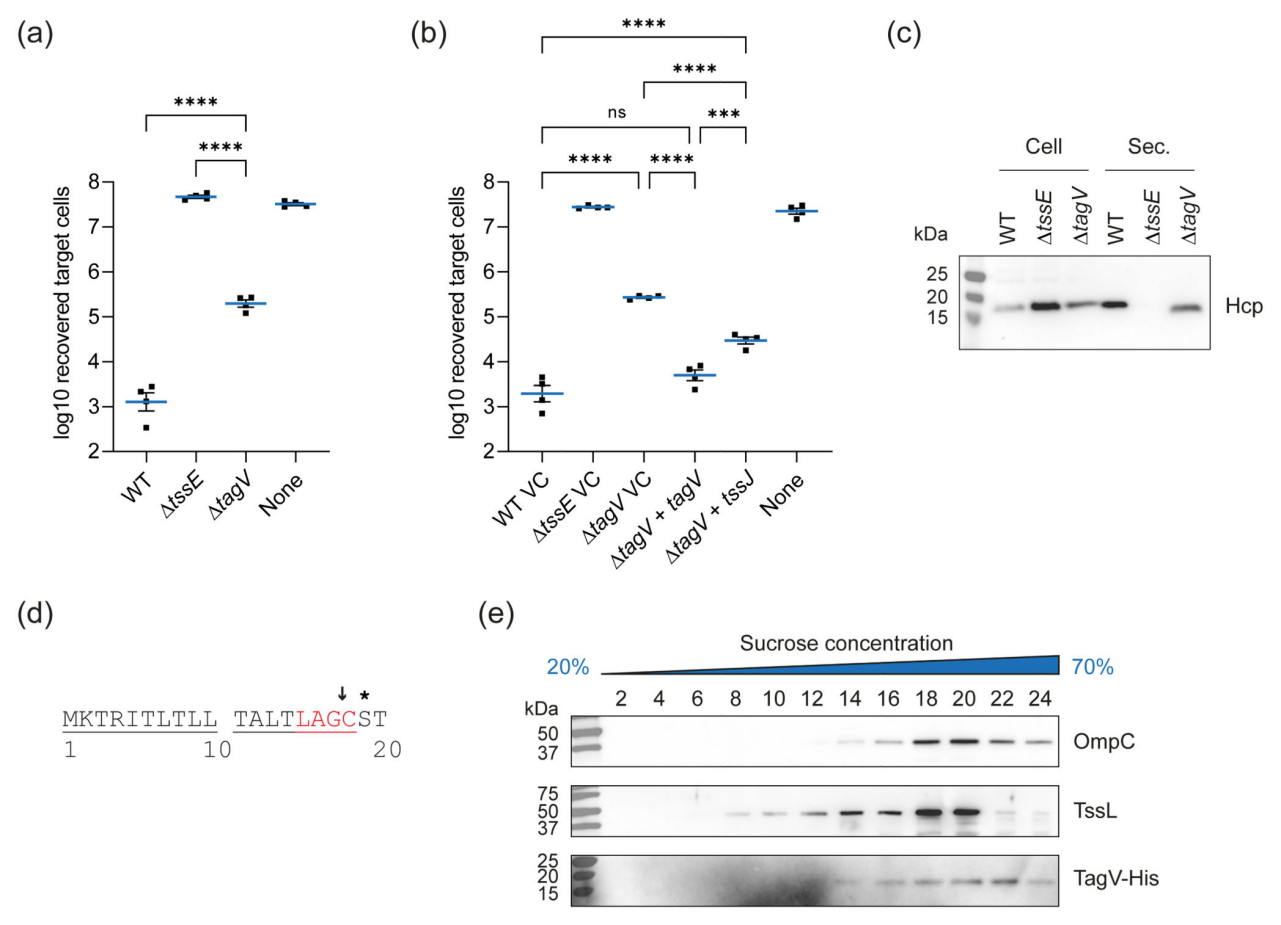
TagV is an outer membrane lipoprotein required for full T6SS activity. (a) T6SS-dependent anti-bacterial activity of wild type (WT) and mutant (Δ*tssE* and Δ*tagV*) strains of *S. marcescens* Db10, as determined by the recovery of *P. fluorescens* KT02 target cells following co-culture with *S. marcescens* at an initial ratio of 1:1 for four hours. (b) Recovery of *P. fluorescens* KT04 following co-culture with WT and mutant strains of *S. marcescens* carrying either the vector control plasmid (pSUPROM, VC) or plasmids directing the expression of *tagV* (+ *tagV*) or *tssJ* (+ *tssJ) in trans*. In panels a and b, data are displayed as mean +/- SEM (n=4) with individual data points overlaid. One-way ANOVA with Tukey’s multiple comparison test was performed (**** *P*<0.0001; *** *P*<0.001; ns, not significant; for clarity, only selected comparisons are displayed). None, sterile media only. (c) Levels of Hcp in the total cellular (Cell) and secreted (Sec.) protein fractions from WT, Δ*tssE* and Δ*tagV* mutant strains of *S. marcescens* as detected by immunoblot. (d) The N-terminus of TagV. The lipoprotein signal peptide is underlined with the lipobox presented in red. The putative cleavage site is indicated by the arrow and the +2 position which determines the inner/outer membrane location of the mature protein is indicated by the asterisk. (e) Inner and outer membrane fractions of *S. marcescens* expressing a His-tagged version of TagV, TagV-His, were separated using a discontinuous sucrose gradient and the presence of OmpC (outer membrane control), TssL (inner membrane control) and TagV in the indicated fractions was detected by immunoblotting with the corresponding antibodies.

TagV is a predicted lipoprotein of 166 amino acids in length that is encoded within the *S. marcescens* Db11 T6SS gene cluster, where it carries the locus tag *SMDB11_2251*. The genomes of *S. marcescens* Db10 and Db11 differ by only one nucleotide therefore the complete genome sequence of Db11 is used interchangeably for Db10 (Iguchi *et al*., 2014). TagV shares no homology with any proteins of known function, however genes encoding homologues of TagV were identified within T6SS gene clusters in several other genera suggesting that the protein plays a conserved role in T6SS activity (Figure 1, Figure S1). Deletion of *tagV* in *S. marcescens* Db10 resulted in a substantial reduction in T6SS-dependent antibacterial activity compared with the wild type strain, as shown by increased recovery of *Pseudomonas fluorescens* following co-culture with the *tagV* mutant compared with wild type *S. marcescens* (Figure 2a). Wild type levels of antibacterial activity were restored when the Δ*tagV* mutant was complemented with a plasmid constitutively expressing *tagV*. In addition, the antibacterial activity of the Δ*tagV* mutant was partially restored through heterologous expression of the core T6SS lipoprotein *tssJ*, suggesting that the role of these two proteins may be linked (Figure 2b). The level of T6SS firing in the Δ*tagV* mutant was determined by using immunoblotting to observe secretion of the expelled component Hcp to the media. This confirmed that there was a reduction of Hcp secretion and thus T6SS firing in the Δ*tagV* mutant, and that Hcp secretion could be fully restored by expression of *tagV in trans* (Figure 2c, Figure S2).

TagV is predicted to be a lipoprotein due to the presence of an 18 amino acid N-terminal signal peptide containing the lipobox “LAGC”. As TagV lacks an aspartic acid at the +2 position of the mature protein, known to promote inner membrane retention (Seydel *et al*., 1999, Yamaguchi *et al*., 1988), it is predicted to be an outer membrane lipoprotein (Figure 2d). Localisation of TagV to the outer membrane was confirmed using a strain of *S. marcescens* carrying a hexahistidine-tagged version of TagV encoded at the normal chromosomal location, denoted *tagV*-His. Total membrane fractions were prepared from *tagV*-His and the inner and outer membranes were separated using a discontinuous sucrose gradient. When the fractions from this density gradient separation were analysed by immunoblotting, the signal from the outer membrane control protein OmpC was prominent within the terminal, high sucrose fractions (22 – 24). In contrast the signal from the inner membrane control protein TssL peaked at a lower sucrose concentration and was at the limit of detection in the terminal fractions. The profile of TagV-His was comparable to that of OmpC, further suggesting that the protein associates with the outer membrane of *S. marcescens* (Figure 2e). The subcellular location of TagV was also investigated by selective detergent solubilisation (Larsen & Biedermann, 1993). However, under these conditions, both TagV-His and the well-characterised outer membrane lipoprotein TssJ (Felisberto-Rodrigues *et al*., 2011) co-purified with the inner membrane control protein TssL (Figure S3). The presence of both TssJ and TagV-His in the solubilised, inner membrane fraction in this experiment suggests that under the conditions utilised, lipoproteins may dissociate from the outer membrane of *S. marcescens*.

### Genetic manipulation of *tagV* results in decreased TssJ production which cannot be restored by complementation with *tagV in trans*

The partial restoration of T6SS-mediated antibacterial activity in the Δ*tagV* strain following heterologous expression of *tssJ* led us to consider that disruption of *tagV (SMDB11_2251)* may affect *tssJ* (*SMDB11_2252*). TssJ production was investigated by immunoblotting and densitometry which revealed a reduction in cellular TssJ levels in the Δ*tagV* strain (Figure S4). To rule out the presence of a ribosome binding site or other sequence required for initiation of TssJ translation within the *tagV* gene, a second Δ*tagV* strain was constructed which retained the C-terminal 15 amino acids of TagV, denoted *tagV*Δ3-151. However, cellular TssJ levels were also reduced in this strain. To determine whether the TagV protein directly influences the production of TssJ, immunoblots were performed using Δ*tagV* and *tagV*_Δ3-151_ carrying a plasmid expressing full length *tagV*. No restoration of TssJ levels was observed under these conditions, suggesting that it is the genetic context of *tagV* that is important for TssJ production (Figure S4). This assertion is supported by data showing that addition of a hexahistidine tag at the C-terminus of *tagV* is also sufficient to reduce TssJ production (Figure S4). This effect is specific to TssJ, as we confirmed that the production of proteins encoded by the genes downstream of *tssJ*, namely TssK, TssL and TssM, was unaffected by deletion of *tagV* (Figure S4f).

It is important to note that we observed full complementation of the original Δ*tagV* mutation back to wild type levels when examining T6SS-dependent antibacterial activity and Hcp secretion upon heterologous expression of *tagV in trans* (Figure 1b, Figure S2). Similarly, we also observed full complementation of the reduced T6SS-dependent antibacterial activity displayed by the *tagV*_Δ3-151_ mutant upon heterologous expression of *tagV in trans* (Figure S5). In this case, however, we did not observe any significant restoration of T6SS activity on heterologous expression of *tssJ*. These data demonstrate that the reduced level of TssJ is not the primary basis for the reduction in T6SS activity in the Δ*tagV* and *tagV*Δ3-151 mutants. In these complementation experiments, TssJ levels are still reduced but T6SS activity is at wild type levels, indicating that this reduced level of TssJ is sufficient to support T6SS activity. Nevertheless, whilst it is clear that TagV is required for full T6SS activity, we cannot exclude the possibility that reduced levels of TssJ may contribute to the phenotype of the *tagV* deletion mutants in some contexts.

### TagV is required for TssJ-independent T6SS firing

**Figure 3:**
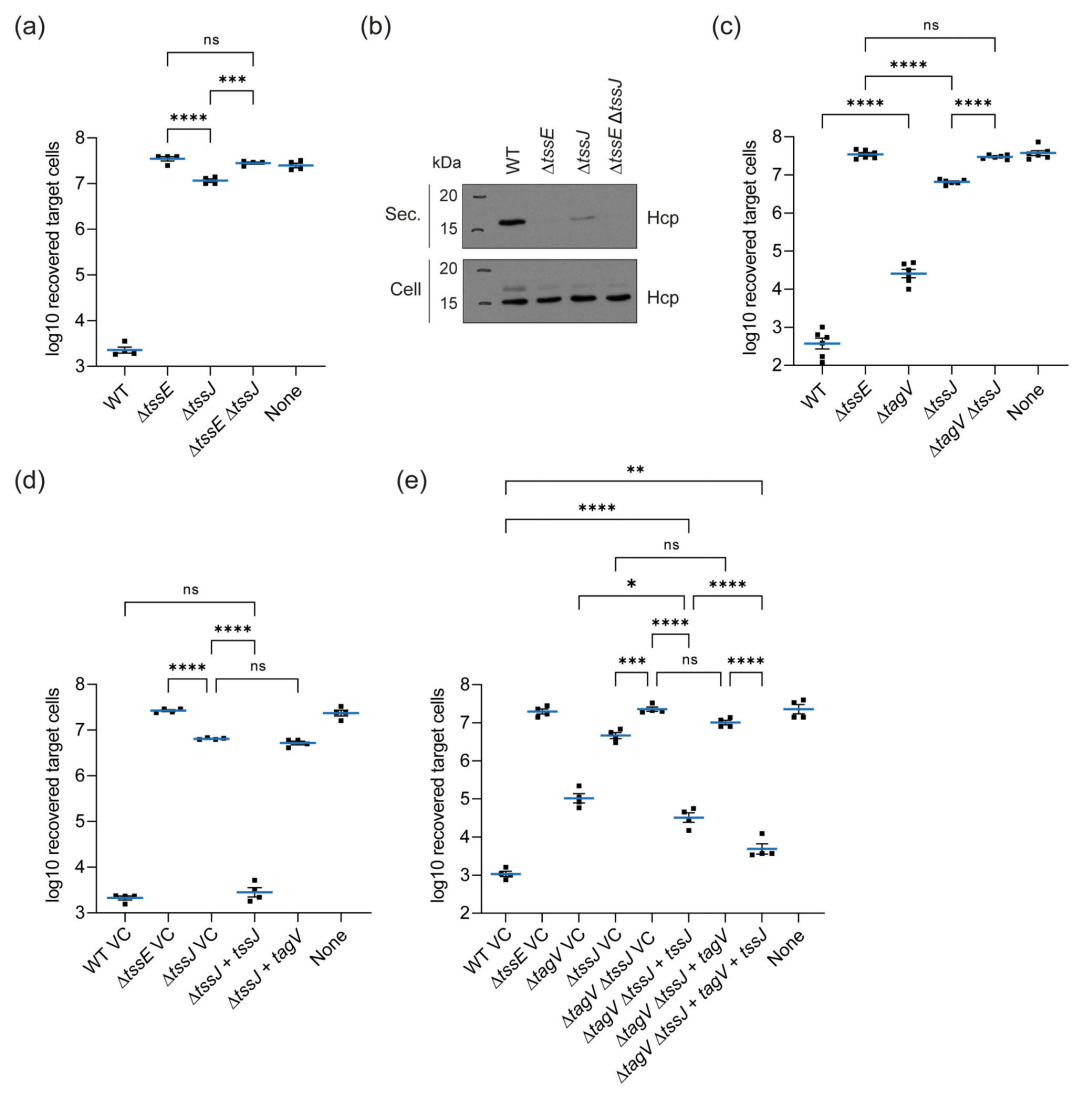
TagV promotes T6SS firing in the absence of TssJ. (a) Recovery of *P. fluorescens* KT02 target cells following co-culture with wild type (WT) and mutant (Δ*tssE, ΔtssJ* and Δ*tssEΔtssJ*) strains of *S. marcescens* at an initial ratio of 1:1 for four hours. (b) Levels of Hcp in the total cellular (Cell) and secreted (Sec.) protein fractions from the indicated strains of *S. marcescens* as detected by immunoblot. (c) Recovery of *P. fluorescens* KT02 target cells following co-culture with WT and mutant (Δ*tagV, ΔtssJ* and Δ*tagVΔtssJ*) strains of *S. marcescens* at an initial ratio of 1:1 for four hours. (d-e) Recovery of *P. fluorescens* KT04 following co-culture with WT and mutant strains of *S. marcescens* carrying either the vector control plasmid (pSUPROM, VC) or plasmids directing the expression of *tagV*(+ *tagV*) and/or *tssJ* (+ *tssJ) in trans*. Quantitative data sets are displayed as mean +/- SEM with individual data points overlaid. One-way ANOVA with Tukey’s multiple comparison test was performed (**** *P*<0.0001; *** *P*<0.001; ** *P*<0.01; * *P*<0.05; ns, not significant; for clarity, only selected comparisons are displayed). None, sterile media only.

The adjacent genes *tagV* and *tssJ* both encode outer membrane lipoproteins that are essential for full T6SS activity, further leading us to hypothesise that the functions of TagV and TssJ may be linked. We previously reported that an in-frame deletion mutant of *tssJ* missing amino acids 3-173 of 176 (*tssJ*_Δ3173_) in Db10 showed a complete loss of T6SS activity. However Db10 *tssJ*_Δ3-173_ could only be partially complemented by expression of *tssJ in trans* (Murdoch *et al*., 2011). Subsequently we discovered that levels of TssK, encoded immediately downstream of *tssJ*, were decreased in this mutant. Therefore we constructed a new mutant of *tssJ* where most of the gene remained intact (*tssJ*_Δ4-28_, herein referred to as Δ*tssJ)* that produces wild type levels of TssK (Figure S6). Using this non-polar mutant, we sought to re-examine the effect of the loss of TssJ on the T6SS dependent antibacterial activity of *S. marcescens*. While the new Δ*tssJ* mutant demonstrated a substantial reduction in antibacterial activity compared with the wild type strain, a small but significant amount of activity was apparent when the mutant was compared with a T6SS-inactive Δ*tssE* strain (Figure 3a). This residual anti-bacterial activity was abolished when *tssJ* was deleted in a Δ*tssE* background, indicating that it is T6SS-dependent. TssJ-independent T6SS firing was confirmed by immunoblotting, which demonstrated a small amount of TssE-dependent Hcp secretion into the culture medium by the Δ*tssJ* mutant (Figure 3b).

To determine if this TssJ-independent T6SS firing required TagV, the antibacterial activity of a Δ*tagVΔtssJ* double mutant was assessed (Figure 3c). The antibacterial activity of this strain was indistinguishable from that of the Δ*tssE* mutant, showing that *tagV* is essential for T6SS firing in the absence of *tssJ*. The T6SS activity of the Δ*tssJ* mutant could be restored through complementation of the mutation by expression of *tssJ* from a plasmid, but not by expression of *tagV*, indicating that the proteins perform different roles during T6SS firing (Figure 3d, Figure S2). Consistent with non-redundant functions for TagV and TssJ, attempts to complement the Δ*tagVΔtssJ* double mutant with plasmid-expressed *tssJ* and/or *tagV* revealed that both genes were required for maximal restoration of antibacterial activity. Whilst the activity of the Δ*tagVΔtssJ* mutant complemented with *tssJ* + *tagV* did not quite reach wild type levels, presumably as a consequence of non-stoichiometric (over)expression of two T6SS components, it was significantly greater than that of Δ*tagVΔtssJ* complemented with *tssJ* alone (Figure 3e). As expected, the Δ*tagVΔtssJ* mutant showed similar activity to the Δ*tssJ* mutant when complemented with *tagV* alone and slightly greater activity than the Δ*tagV* mutant when complemented with *tssJ*, the latter consistent with the single Δ*tagV* mutant expressing *tssJ in trans*.

### TagV does not interact with TssM or TssL under experimental conditions

**Figure 4:**
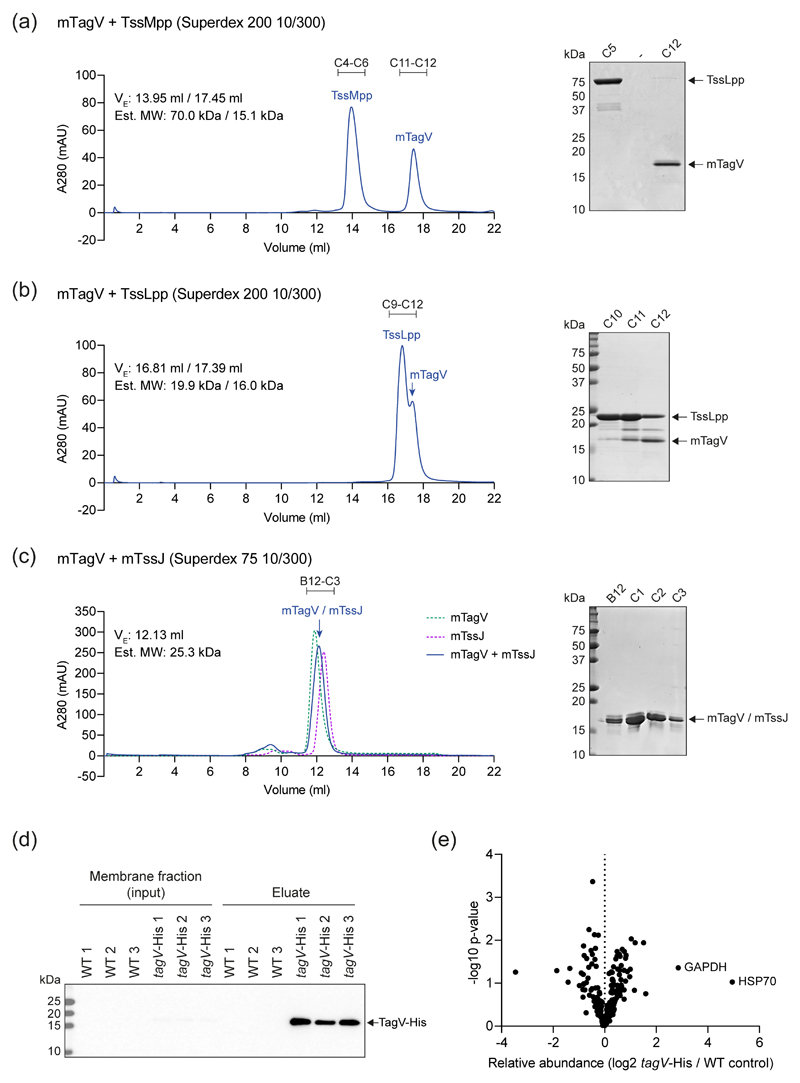
TagV does not interact with TssL, TssM or TssJ. (a-c) Purified mature TagV protein (mTagV) was combined with the purified periplasmic domains of TssM (TssMpp, panel a) or TssL (TssLpp, panel b), or with mature TssJ (mTssJ, panel c), and the samples were separated by size exclusion chromatography using the column indicated. The elution volume (Ve) and estimated molecular weight (Est. Mw) of each peak, based on calibration of the column with protein standards, is noted in each case. Protein fractions corresponding to peaks in the chromatograms were visualised by SDS-PAGE and Coomassie staining. In panel c, since mTagV and mTssJ have similar molecular weights and cannot be separated by SDS-PAGE, the corresponding chromatogram for each protein alone is overlaid (dotted lines) to confirm that the peak observed for the combined sample does not display an altered retention compared with the individual proteins. (d-e) Attempt to identify TagV-associated proteins using an *in vivo* co-purification approach. Total membrane fractions prepared from wild type (WT) and *tagV*-His strains of *S. marcescens* were incubated with Ni^2+^ beads and the eluates were probed for the presence of TagV-His by immunoblot (d) or analysed by quantitative mass spectrometry (e). In panel e, no proteins were identified as being significantly enriched in the *tagV*-His samples compared with the WT control. Significant enrichment was defined as relative abundance in *tagV*-His/control > 4-fold with p-value <0.05 (n=3 biological replicates). Proteins with the highest average fold change values in the *tagV*-His samples (GAPDH and HSP70) are labelled. TagV was identified in all three *tagV*-His samples (sequence coverage 21-36%; unique peptides 4-8) and in none of the control samples (and therefore is not in the plot in panel e due to lack of a fold change value).

The cellular location of TagV and the ability of the protein to drive T6SS firing in the absence of TssJ led us to hypothesise that TagV could interact with the TssL or TssM subunit of the MC. To investigate this, recombinant, mature (m)TagV and the periplasmic domains of TssL (TssLpp) and TssM (TssMpp) were purified to apparent homogeneity by Ni^2+^ affinity chromatography and size exclusion chromatography (SEC) (Figure S7). The ability of mTagV to form a complex with TssLpp or TssMpp was assessed by separating equimolar mixtures of each pair of proteins using SEC. No higher molecular weight / smaller elution volume peaks corresponding to mTagV in complex with TssLpp or TssMpp were observed, suggesting that mTagV does not bind to TssLpp or TssMpp under the conditions used (Figure 4a-b). We also investigated whether mTagV can interact with mature TssJ (mTssJ) using the same approach. This showed that TagV does not interact with TssJ *in vitro* (Figure 4c), consistent with a role independent of TssJ.

We also attempted to identify T6SS-associated or other protein binding partners for TagV using a co-purification (“pulldown”) approach. Total membrane protein fractions prepared from wild type Db10 (control), and the *tagV*-His strain were incubated with Ni^2+^ beads to isolate TagV-His together with any interacting proteins. The protein content of the resulting eluates was analysed by label-free quantitative mass spectrometry. While TagV-His was identified in the eluates by immunoblotting and mass spectrometry, no co-eluting binding partners, defined as proteins only present or significantly enriched in the *tagV*-His eluates compared with the controls (increase in abundance ≥ 4x, p < 0.05), were identified by quantitative label-free mass spectrometry (Figure 4d-e). Additionally, to exclude an interaction between TagV and TssJ being missed due to lower levels of TssJ in the *tagV*-His strain, we performed a pull-down in a strain overexpressing both TagV-His and TssJ from a plasmid. However no co-purification of TssJ with TagV-His could be detected (Figure S8).

### The predicted structure of TagV

**Figure 5:**
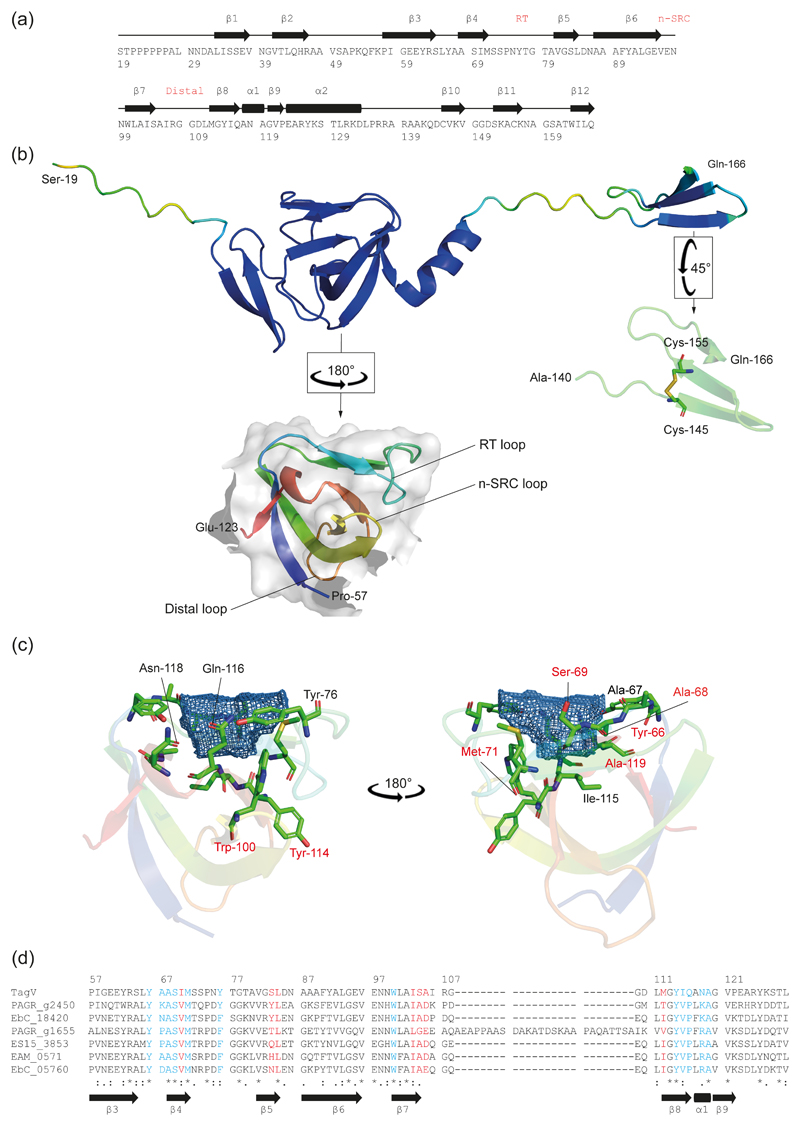
The predicted structure of TagV. Sequence (a) and ribbon diagram (b) representations of the predicted structure of the mature TagV protein. In panel b, the full length protein is coloured based on the reported confidence of the AlphaFold modelling, from red (pLDDT<50, lowest confidence) to blue (pLDDT>90, highest confidence). The predicted SH3b domain (P57–E123) has been reproduced and rotated to highlight the position of the RT, n-Scr and distal loops and is coloured from blue (N-terminus) to red (C-terminus). The putative C-terminal domain (A140–Q166) has been reproduced and rotated to highlight the disulphide bond (yellow). (c) The predicted ligand binding pocket, P0, identified using the DogSiteScorer algorithm (blue mesh). Residues involved in formation of the pocket are labelled and displayed as sticks. Residues labelled in red are conserved amongst the TagV homologues in panel d. (d) Multiple sequence alignment of the putative SH3b domain in TagV and the six TagV homologues presented in Figure 1. Residues involved in formation of pocket P0 and P1 are highlighted in blue and red respectively.

A structural prediction for TagV was generated using AlphaFold2 (Jumper *et al*., 2021) which suggested that mature protein is composed of twelve β strands and two α helices (Figure 5a-b). The predicted mTagV protein contains two domains that were folded with high confidence (defined as a pLDDT (Mariani *et al*., 2013) greater than 90): an SH3b like domain (P57–E123), and a small, C terminal domain (A140–Q166) comprising three short β sheets which are stabilised by a disulphide bond between C145 and C155 (Figure 5b). This disulphide bond is consistent with previous data demonstrating that TagV is subject to DsbA-dependent oxidation, implying the presence of an intramolecular disulphide bond (Mariano *et al*., 2018).

Typical eukaryotic SH3 domains consist of five β strands connected by three loops (denoted RT, N-Src and distal) and a short 310 helix. A hydrophobic binding surface facilitates promiscuous engagement of proline-rich helices, while the specificity of individual SH3 domains is determined by a “specificity site” which is flanked by the RT and n-SRC loops. In most cases, the specificity site is negatively charged and interacts with the positively charged residues located at either side of the target PxxP consensus (Teyra *et al*., 2017). Bacterial SH3 (SH3b) domains share a similar tertiary structure to eukaryotic SH3 domains despite a low degree of sequence similarity (Shan *et al*., 2021).

The putative SH3b domain of TagV includes a long RT loop encompassing parallel β sheets and a short 3_10_ helix at the extreme C-terminus of the domain (Figure 5b). Analysis using Dali (Holm, 2020) revealed that the TagV SH3b domain has substantial homology with several SH3b domains involved in peptidoglycan binding, raising the possibility that TagV may interact with the *S. marcescens* cell wall (Table 1). Prospective ligand binding pockets were visualised using the DoGSiteScorer algorithm which has previously been used to identify the ligand binding pockets of an SH3b domain from *Clostridium perfringens* autolysin CpAcp (Psm, Shan *et al*., 2021). The results indicated the presence of two pockets, denoted P0 and P1, with volumes of 144 Å^3^ and 100 Å^3^, respectively (Figure S9a). Pocket P1 is contained within a hydrophobic groove and thus is analogous to the peptide binding domain of canonical SH3 domains (Figure S9b). Pocket P0 is flanked by the RT loop suggesting it represents the specificity site. However, unlike in canonical SH3 domains, in TagV this putative pocket carries a net positive charge (Figure 5c, Figure S9c). Similar SH3b pockets have previously been implicated in peptidoglycan binding by Psm, based on its interaction with an acetic acid molecule, however this is yet to be confirmed experimentally (Shan *et al*., 2021). Multiple sequence alignment of TagV and its homologues revealed a substantial degree of sequence conservation in the regions of the protein sequence that comprise the P0 pocket suggesting that these regions may be important for the function of TagV (Figure 5c-d). In contrast, limited sequence conservation was apparent amongst the residues involved in P1 pocket formation.

### A conserved tryptophan residue contributes to but is not essential for TagV activity

**Figure 6:**
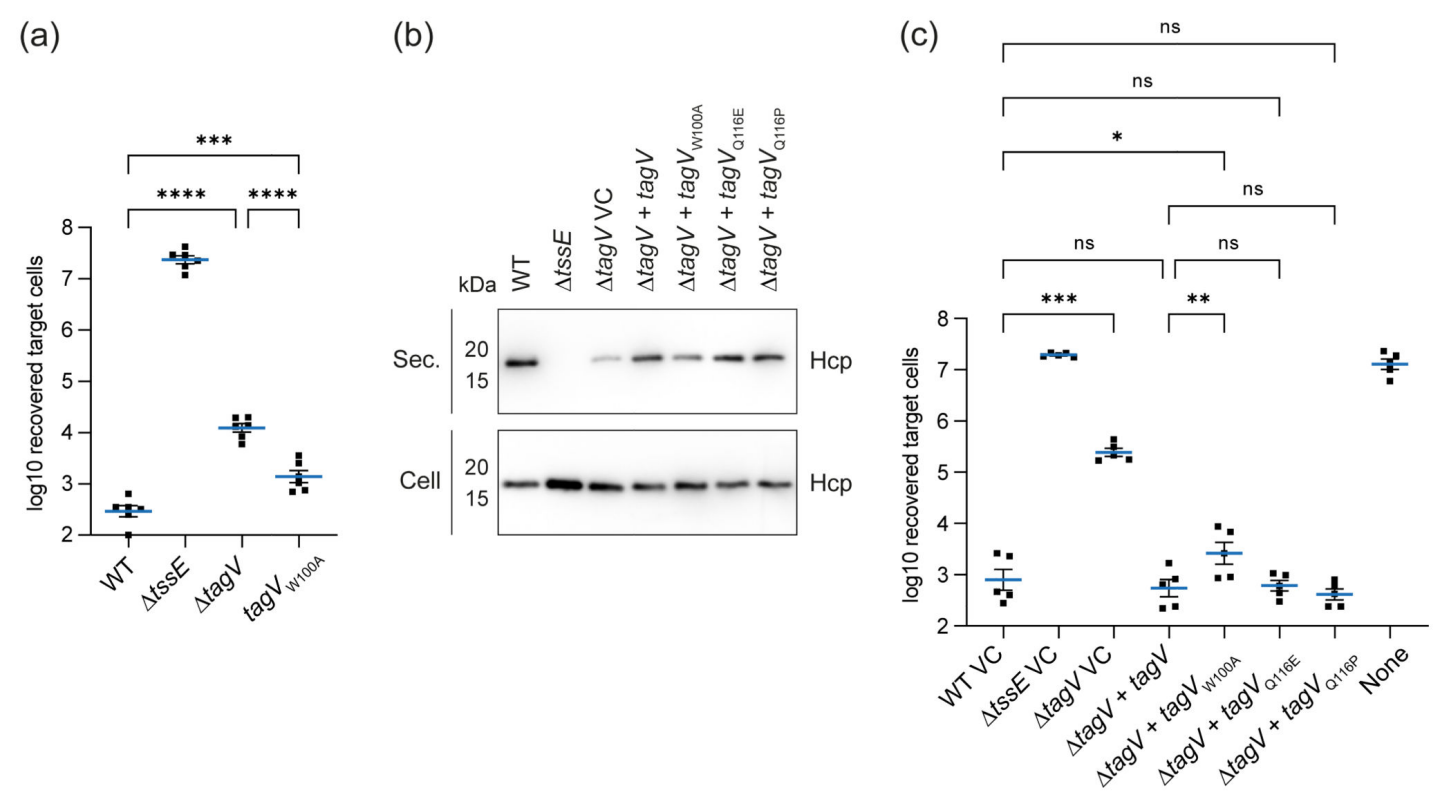
Introduction of a W100A mutation in TagV causes a reduction in T6SS activity. (a) Recovery of *P. fluorescens* KT02 target cells following co-culture with wild type (WT) and mutant (Δ*tssE*, Δ*tagV* and *tagV*_W100A_) strains of *S. marcescens* at an initial ratio of 1:1 for four hours. These data form part of the larger experiment and analysis depicted in Figure S4d. (b) Levels of Hcp in the total cellular (Cell) and secreted (Sec.) protein fractions from strains of *S. marcescens* as detected by immunoblot. WT and mutant strains of *S. marcescens* carried either the vector control plasmid (pSUPROM, VC) or plasmids directing the expression *in trans* of native *tagV* (+ *tagV*) or point mutants (+ *tagV*_W100A_, *togV*_Q116E_ or *tagV*_Q1116P_). (c) Recovery of *P. fluorescens* KT02 following co-culture with strains of *S. marcescens* as indicated. Data are displayed as mean +/- SEM with individual data points overlaid (n=5). Repeated measures one-way ANOVA with Tukey’s multiple comparison test was performed (*** *P*<0.001; ** *P*<0.01; * *P*<0.05; ns, not significant; for clarity, only selected comparisons are displayed). None, sterile media only.

In previous studies of SH3b domain-containing proteins, mutation of a highly conserved tryptophan located within the SH3 ligand binding site was sufficient to abolish interaction with proteins and peptidoglycan (Gonzalez-Delgado *et al*., 2020, Lu *et al*., 2006, Wunderlich *et al*., 1999). The SH3b domain of TagV contains a single tryptophan within P0 (W100) which is conserved amongst the TagV homologues from other genera (Figure 5c-d), leading us to hypothesise that mutation of this residue may result in a loss of TagV activity. A mutant strain of *S. marcescens* encoding an alanine in place of W100 in TagV was generated. This strain, *tagV_W100A_*, does not phenocopy a *tagV* mutant, but instead displays a modest reduction in T6SS activity compared with its wild type parent (Figure 6a). Similarly, when the ability of the *tagV_W100A_* variant to complement the reduced T6SS activity of Δ*tagV* mutant when expressed *in trans* was compared with that of the wild type, we observed that it appeared less able to restore Hcp secretion and showed significantly reduced anti-bacterial activity (Figure 6b-c). By comparing the TagV structure prediction with the interactions between Psm and acetic acid (Shan *et al*., 2021) and between lysostaphin and a peptidoglycan-like ligand (Gonzalez-Delgado *et al*., 2020), we predicted that glutamine 116 of TagV was likely to interact with the TagV ligand. Interestingly the position occupied by Q116 in TagV is occupied by a proline in all the other TagV homologues we examined (Figure 5d). In order to test the contribution of Q116 to TagV function, we tested the ability of TagV variants Q116E and Q116P, encoded by *tagV*_Q116E_ and *tagV*_Q116P_ respectively, to complement the reduced T6SS activity of the Δ*tagV* mutant when expressed *in trans*. This showed that there was no detectable difference between *tagV*_Q116E_ or *tagV*_Q116P_ and wild type *tagV*, with all three able to fully restore T6SS-dependent antibacterial activity to wild type levels (Figure 6b-c). These data indicate that the conserved tryptophan at position 100 of TagV, but not the glutamine or proline at position 116, is required for full T6SS activity, and are consistent with a role for the conserved W100 in interaction with peptidoglycan or other ligand of TagV. However, loss of a single amino acid side chain may have limited impact in the context of a network of multiple interactions between TagV and its ligand. Detailed further studies will be required to define the ligand and mode of binding for the SH3 domain of TagV.

## Discussion

The T6SS is a widely-occurring and versatile bacterial weapon. While the main structure is composed of fourteen highly conserved core proteins that are essential for T6SS function, the firing behaviour of many systems is influenced by its complement of T6SS accessory proteins (Tag proteins) that vary from species to species and system to system. A summary of the Tag/T6SS accessory proteins described to date and their proposed or demonstrated functions during T6SS deployment is provided in Table S1. Peptidoglycan binding appears to be a common property of the Tag proteins, although several of the putative peptidoglycan binding Tag proteins reported so far have no experimentally demonstrated function. Here, we have identified a novel T6SS accessory protein that is required for optimal T6SS firing in *S. marcescens* and likely also binds peptidoglycan. Based on the T6SS accessory function of the protein and its presence in the T6SS cluster of several species and genera, we have named this protein “TagV” in line with the nomenclature established by Shalom and colleagues (Shalom *et al*., 2007).

TagV is a lipoprotein that is predicted to contain an SH3b domain structurally homologous with the SH3b domain of the phage-associated endolysin Psm (Tamai *et al*., 2014) and several other peptidoglycan binding proteins (Table 1). Perhaps the most well-characterised SH3b-peptidoglycan interaction comes from crystal studies performed using *Staphylococcus aureus* lysostaphin in complex with a ligand corresponding to a peptidoglycan tetrapeptide with a pentaglycan lateral chain (P4-G5) (Gonzalez-Delgado *et al*., 2020). Binding of the tetrapeptide stem of P4-G5 by lysostaphin is partially achieved via a positively charged pocket which engages with the carboxylate group of the terminal alanine. A similar pocket was predicted to engage with the peptide side chain of peptidoglycan in Psm, based on its interaction with a bound acetic acid molecule sequestered from the reservoir solution (Gonzalez-Delgado *et al*., 2020, Tamai *et al*., 2014). The ribbon structure of Psm (PDB 4krt) and the surface of the putative SH3 domain of TagV are superimposed in Figure S10, with a root mean square deviation (RMSD) of 0.719 Å. According to this, the position of the sequestered acetic acid molecule would correspond to a position within the P0 pocket of TagV, perhaps indicating a role for this pocket in TagV-peptidoglycan engagement. In lysostaphin, tetrapeptide binding requires clustering of SH3b domains, which facilitates engagement of a second, hydrophobic pocket at the opposite side of the molecule (Gonzalez-Delgado *et al*., 2020). Given the position of the TagV P1 pocket, within a hydrophobic groove on the opposite side of the SH3b domain, it is tempting to speculate that a similar binding mechanism, involving clustering of TagV, is responsible for peptidoglycan engagement during T6SS assembly. However, this is yet to be determined experimentally.

Based on the cellular location of TagV and the effect of its deletion on T6SS activity, we propose that the protein is required to stabilise the T6SS through interactions with the peptidoglycan cell wall and/or outer membrane. No T6SS-associated binding partners could be identified by the *in vivo* co-purification approach used in this study. However, particularly given its potential association with both the outer membrane and the cell wall, we believe that it is likely that TagV-T6SS interactions only occur in the context of a fully or partially assembled T6SS in an intact cell, and are lost following cell lysis and protein solubilisation. Alternatively, peptidoglycan-dependent clustering of TagV proteins may be an essential prerequisite for interaction of TagV with another T6SS protein(s). Our data suggest that TssJ is the primary link between the T6SS and the outer membrane in *S. marcescens*. However, it is interesting to note that TssJ is only essential for T6SS function in the absence of TagV, and that maximum T6SS efficiency was only observed when both proteins were present. The suggestion that TssJ is not the only way to connect the T6SS and the outer membrane is consistent with the fact that members of the *Acinetobacter* genus do not contain a TssJ homologue despite the presence of a functional, and presumably outer membrane associated, T6SS (Cherrak *et al*., 2019).

Based on our findings, we propose that TagV and TssJ promote T6SS assembly and function through distinct mechanisms and are unlikely to directly interact with each other. The observation that TssJ-independent T6SS activity depends on TagV (and *vice versa)*, and that *tagV* mutants show impaired function even when TssJ is overexpressed (and *vice versa)*, suggests two independent but complementary means to anchor or stabilise the T6SS. It remains to be determined whether the observation that deletion of the *tagV* gene causes a reduction in the levels of TssJ, specific to that protein, reflects a regulatory interaction or an experimental artefact. In terms of whether this reduction in TssJ is partly contributing to the phenotype of our *tagV* mutants, two possibilities can be explored. The first is that the *tagV* mutant phenotype does include a contribution from reduced TssJ, but full complementation by expression of *tagV in trans* reflects promotion of the alternative TagV-dependent assembly pathway to compensate for reduced TssJ. However overexpression of *tagV* does not significantly restore function in the Δ*tssJ* mutant. The second possibility is that the *tagV* mutant phenotype does not include any contribution from reduction of TssJ but rather, in some contexts, overexpression of TssJ can promote the TssJ-dependent pathway to partially compensate for loss of TagV. Both of these explanations are fully consistent with our model that TssJ and TagV contribute to T6SS function through independent, alternative modes.

In summary, we have identified a novel T6SS accessory protein (TagV) that is conserved amongst several species and is required for full T6SS activity in *S. marcescens*. In the absence of the T6SS core component TssJ, TagV is necessary and sufficient to stimulate a modest amount of Hcp secretion and T6SS-mediated anti-bacterial activity. We propose that TagV stabilises the T6SS by connecting the assembled structure to the peptidoglycan cell wall, however further studies will be required to determine the T6SS-associated binding partner(s) of TagV and its exact role during T6SS assembly.

## Experimental Procedures

### Bacterial strains and growth conditions

The strains used in this study are listed in Table S2 and were routinely cultured in low salt LB (tryptone 10 g/l, yeast extract 5 g/l, NaCl 5 g/l) at 30°C with agitation. Streptomycin, kanamycin and ampicillin/carbenicillin were routinely used at 100 μg/ml. Mutants were constructed in *S. marcescens* Db10 using the pKNG101 suicide vector as previously described (Murdoch *et al*.,2011). The plasmids used for recombinant protein production were derived from pET-15b (Novagen) and are listed in Table S2. The primers used for plasmid generation and mutagenesis are listed in Table S3. Site directed mutagenesis was performed using The Q5^®^ Site-Directed Mutagenesis Kit (New England Biolabs) according to the manufacturer’s instructions.

### Bacterial co-culture (competition) assays for T6SS dependent antibacterial activity

Competition assays were performed as described previously (Murdoch *et al*., 2011). Briefly, attacker and target cells were adjusted to an OD_600_ of 0.5 in low salt LB and combined 1:1. The resulting suspensions were spotted onto pre-warmed LB agar plates in 25 μl aliquots and incubated at 30°C for 4 h. Surviving target cells were enumerated by serial dilution and plating onto selective LB agar.

### SDS-PAGE and immunoblotting

In SDS-PAGE experiments, proteins were combined with 4x protein loading dye (200 mM Tris-HCl pH 6.8, 6.4 % SDS, 6.4 mM EDTA, 32 % glycerol, 0.07% bromophenol blue) and heated to 100°C for 5 min. 10 μl aliquots of the prepared samples were separated on 15 % Tris-HCl or 10 % Tris-tricine gels and stained with Instant Blue (Expedeon). For immunoblotting, gels were transferred onto PVDF membrane, blocked with 2.5 % milk powder (Marvel) in PBS + 0.1 % Tween-20 and probed with polyclonal rabbit anti-Hcp (1:6000), anti-TssJ (1:2000) or anti-TssK (1:2000) antiserum. Bound antibodies were detected using a 1:10,000 dilution of HRP-conjugated goat anti-rabbit IgG and an enhanced chemiluminescence detection kit (Millipore). Signal detection was performed using the Azure 600 imaging system (Cambridge bioscience).

For TssJ quantification by densitometry, strains were adjusted to an OD_600_ of 0.25 in low salt LB, spotted onto pre-warmed LB agar plates in 25 μl aliquots and incubated at 30°C for 4h. The resulting cellular material was lysed in 250 μl aliquots of Bugbuster protein extraction reagent (Sigma-Aldrich) and adjusted to 400 μg/ml using a Pierce Coomassie Plus (Bradford) Assay Kit (Thermo-Fisher) according to the manufacturer’s instructions. 2 μg aliquots were separated by SDS-PAGE and analysed by immunoblotting using anti-TssJ antiserum as outline above. Densitometry was performed using the Azure spot software package (Cambridge Bioscience).

### Inner / outer membrane fractionation

Membrane fractionation experiments were performed essentially according to the protocols of Larsen and Biedermann(Larsen & Biedermann, 1993). Briefly, cultures of *tagV*-His were grown in 25 ml LB for 7 h at 30°C from a starting OD_600_ of 0.02. Cells were recovered by centrifugation and then resuspended in 2 ml of 20% sucrose in 10 mM HEPES pH 7.4 and incubated on ice for 5 min. Spheroplasts were formed by rapidly mixing 2 ml of spheroplast buffer (10 mM HEPES pH 7.4, 20 mM EDTA, 300 μg/ml lysozyme) with the sample and incubation overnight on ice. Spheroplasts were collected by centrifugation at 20,000 xg, resuspended in 3.6 ml of 10 mM HEPES pH 7.4 and lysed by sonication using a 2mm microtip (amplitude 20%, 6 pulses of 15 sec separated by 30 sec intervals). The cellular debris was removed by centrifugation at 5000 xg and the lysate was 0.2 μm filtered and centrifuged at 40,000 rpm for 30 min at 4°C using a TLA-120.2 fixed angle rotor (r_av_ approx. 57,000 xg). The pellet, containing the total membrane fraction, was resuspended in 500 μl of 10 mM HEPES pH 7.4.

Discontinuous sucrose gradients were prepared by sequential layering of nine sucrose solutions (30%, 35%, 40%, 45%, 50%, 55%, 60%, 65%, 75%) buffered with 10 mM HEPES pH 7.4 and allowed to stand at 4°C overnight. 400 μl of the total membrane fraction was gently loaded onto the top of the pre-formed gradient and the samples were ultracentrifuged at 40,000 rpm for 24 h at 4°C using an SW41-Ti swinging bucket rotor (r_av_ approx. 197,000 xg). 24 fractions were aspirated and analysed by immunoblotting as above, using polyclonal rabbit anti-TssL antiserum (1:2000), rabbit polyclonal anti-OmpC IgG (1:5000, Invitrogen) or mouse anti-His IgG (1:5000, Qiagen).

For selective detergent solubilisation 100 μl of the total membrane fraction was combined with 240 μl of 2.75 % sarkosyl in 10 mM HEPES pH 7.4 and incubated at RT for 1 h. The outer membrane fraction was recovered by centrifugation at 40,000 rpm for 30 min at 4°C using a TLA 120.2 rotor (r_av_ approx. 57,000 xg) and the supernatant, containing the inner membrane fraction was collected. The outer membrane pellet was washed by three cycles of resuspension in 2% sarkosyl and centrifugation as above and solubilised in 350 μl of 1% DDM in PBS.

### Recombinant protein production and size exclusion chromatography

The periplasmic domain of TssM (TssMpp, L527:P1211) and mature TagV (mTagV, S19:Q166) were expressed from pET15b-TEV in SHuffle T7 cells through overnight incubation at 16 °C in the presence of 0.5 mM IPTG. TssL (TssLpp, G218:K406) was expressed from pSC102 (English *et al*., 2014) under identical conditions. The cells were collected by centrifugation at 5000 xg and resuspended in 1:100^th^ volume of lysis buffer (50 mM Tris-HCl pH7.5, 0.5 M NaCl, 20 mM imidazole) supplemented with complete EDTA-free protease inhibitor cocktail (Millipore). Cell lysates were prepared by pressure cell lysis at 15,000 psi and clarified by centrifugation at 48,000 xg and 0.45 μm filtration. Recombinant proteins were purified using a three-step isocratic imidazole elution (60 mM, 120 mM and 200 mM) and a 5 ml Histrap HP column. Proteins were further purified by size exclusion chromatography (SEC) using HiLoad S200 (TssMpp and TssLpp) and S75 (mTagV) 16/600 columns, snap frozen and stored at -80°C. Mature TssJ (mTssJ) was produced as described previously (Rao *et al*., 2011) with minor modifications.

### Analysis of protein complex formation by size exclusion chromatography

Recombinant mTagV was combined in a 1:1 molar ratio with recombinant TssLpp (130 μM each protein) or TssMpp (90 μM each protein) in 50 mM Tris (pH7.5), 150 mM NaCl in a final volume of 100 μl. The sample was incubated at room temperature for 15 min prior to injection onto a Superdex 200 10/300GL Increase column. For combination of mTagV with mTssJ, recombinant proteins purified by Ni^2+^ affinity chromotography were combined in a 1:1 molar ratio (170 μM each protein) and then 250 μl of the mixture separated on a Superdex 75 10/300GL column in 50 mM Tris (pH8.0), 100 mM NaCl, 2 mM β-mercapthoethanol.

### Co-purification experiments

For the co-purification (‘pull-down’) experiment using chromosomally-encoded TagV-His, cultures of wild type *S. marcescens* Db10 and *tagV*-His were grown in 100 ml LB for 7 h at 30°C from a starting OD_600_ of 0.02. Cells were recovered by centrifugation and resuspended in 16 ml of PBS supplemented with complete EDTA-free protease inhibitor cocktail. Cell lysates were prepared by sonication using a ½ inch tip (amplitude 50%, 6 pulses of 15 sec separated by 30 sec intervals) and clarified by centrifugation at 48,000 xg and 0.45 μm filtration. The supernatant was subjected to ultracentrifugation at 45,000 rpm for 2 h at 4°C using a Type 50.2 Ti Rotor (r_av_ approx. 184,000 xg). The pellet, containing the total membrane fraction, was solubilised in 2 ml of 1% n-dodecyl-β-D-maltoside in PBS for 1 h at RT and then diluted 1:4 with PBS prior to the addition of 30 μl of magnetic Ni-NTA beads (Qiagen) and incubation overnight at 4°C with gentle agitation. The beads were washed three times with wash buffer (50 mM sodium phosphate buffer pH7.4, 500 mM NaCl, 0.1 % DDM) using a magnetic rack and the bound proteins were eluted using 50 μl of elution buffer (50 mM sodium phosphate buffer pH7.4, 500 mM NaCl, 0.1 % DDM, 250 mM imidazole).

For the co-purification experiment using plasmid-based expression of TagV-His and TssJ, cultures of *S. marcescens* Db10 Δ*tagVΔtssJ* carrying pSC2017 or pSC2040 were grown in 50 ml LB + Kan for 5 h at 30°C from a starting OD600 of 0.025. Cells were recovered by centrifugation and resuspended in 2 ml of 50 mM Tris-HCl pH 8.0. Cell lysates were prepared by sonication (as above, 12 pulses) and clarified by centrifugation at 15,000 rpm for 20 min at 4°C (benchtop centrifuge). The supernatant was subjected to ultracentrifugation at 80,000 rpm for 30 min at 4°C using a TLA 120.2 rotor (r_av_ approx. 227,000 xg). The pellet, containing the total membrane fraction, was resuspended in 50 mM Tris-HCl pH 8.0, 100 mM NaCl, 2% DDM, 20 mM imidazole and then added to 30 μl of washed magnetic Ni-NTA beads (Qiagen) and incubated for 90 min at room temperature with gentle agitation. The beads were washed three times with wash buffer (20 mM Tris-HCl pH 8.0, 100 mM NaCl, 50 mM imidazole, 0.1% Triton X100) and the bound proteins were eluted using 30 μl of 2x SDS-PAGE sample buffer and heating to 100°C for 2 min.

### Analysis of co-purification eluate samples by mass spectrometry

Eluted protein samples were digested with 2.5 μg trypsin overnight and processed using S-Trap Micro columns (Protifi). Digested peptides were analysed on a Q-Exactive Plus instrument (Thermo Scientific) coupled to a Dionex Ultimate 3000 HPLC system (Thermo Scientific) using a 1.6-28% gradient of acetonitrile in 0.1 % formic acid. For each MS1 scan, the ten highest intensity peaks from a mass range of 350-1600 m/z (resolution = 70,000) were then taken forward for MS2 analysis (resolution = 17,500). Spectra were fragmented using Higher-energy C-trap dissociation (HCD). Label-free analysis was performed using MaxQuant version 1.6.2.10 (Tyanova *et al*., 2016a). Data analysis was performed using Perseus version 1.6.12.0 (Tyanova *et al*., 2016b).

### Protein structural predictions

The predicted structure of mTagV was generated using AlphaFold2 hosted on Google Colab (Jumper *et al*., 2021). Structural homologs of the predicted SH3b domain (P57-E123) were identified using the Dali server (Holm, 2020) and ligand binding pockets were identified using the DoGSiteScorer algorithm hosted on the University of Hamburg website (Volkamer *et al*., 2012). Structural representations were generated using the PyMOL Molecular Graphics System, Version 2.0 (Schrödinger).

## Supplementary Material

Supplementary Data

## Figures and Tables

**Table 1:** Structural homologues of the TagV SH3 domain. Homologues were identified by analysing the predicted structure of the SH3 domain (P57-E123) of TagV against the PDB_25_ database using Dali (Holm, 2020) and including matches with RSMD < 2Å and Z-score > 5.

PDB entry	Chain	Z-score	RMSD	% ID	PDB title
4krt	A	10.2	1.7	10	X-ray structure of endolysin from *Clostridium perfringens* phage phiSM101
6bim	A	9.6	1.8	15	Structure of NlpC1 from *Trichomonas vaginalis*
6sqx	A	8.2	1.6	13	Insights into a novel NlpC/P60 endopeptidase from *Photobacterium damselae* subsp. *piscicida*
5udn	B	7.5	1.8	10	Phage-associated cell wall hydrolase PlyPy from *Streptococcus pyogenes*, space group P3121
5d74	B	7.4	1.9	16	The crystal structure of Ly7917
6ilu	A	6.9	1.9	20	Endolysin LysPBC5 CBD
3fc3	A	5.9	1.6	2	Crystal structure of the beta-beta-alpha-Me type II restriction endonuclease Hpy99I
2y35	A	5.6	1.7	6	Crystal structure of Xrn1-substrate complex
