# Supplementary material for: The accessory protein TagV is required for full Type VI secretion system activity in Serratia marcescens
Source: Mol Microbiol. Author manuscript; Available in PMC 2023 Jul 23. (PMC7614798; doi:10.1111/mmi.15027)
Supplement: Supplementary Data [file EMS180548-supplement-Supplementary_Data.pdf]

Supplementary Information

The accessory protein TagV is required for full Type VI secretion system activity in *Serratia marcescens*

Mark Reglinski[1†], Laura Monlezun[1,2†] and Sarah J. Coulthurst[1]*

[1] Division of Molecular Microbiology, School of Life Sciences, University of Dundee, Dundee, United Kingdom DD1 5EH.

[2] Current address: Université Paris Cité, CNRS, Expression Génétique Microbienne, Institut de Biologie Physico-Chimique, Paris, France.

† These authors contributed equally

* For correspondence: Sarah J. Coulthurst (s.j.coulthurst@dundee.ac.uk)

List of contents

Figure S1. Multiple sequence alignment of TagV and its homologues.

Figure S2. The reduced Hcp secretion observed in the Δ*tagV* and Δ*tssJ* mutants can be complemented by expression of the respective gene *in trans*.

Figure S3. The outer membrane lipoproteins TagV and TssJ solubilise with the inner membrane of *S. marcescens* in selective detergent solubilisation experiments.

Figure S4. Genetic manipulation of *tagV* results in decreased TssJ production which cannot be restored by complementation with *tagV*.

Figure S5. Expression of *tagV* but not *tssJ in trans* is able to restore T6SS activity in the $tagV_{\Delta3\text{-}151}$ mutant.

Figure S6. TssK production in strains carrying two different deletions in *tssJ*.

Figure S7. Integrity of proteins used for *in vitro* complex formation experiments.

Figure S8. TssJ does not co-purify with TagV-His when both are expressed from a plasmid.

Figure S9. The predicted surface topology of the putative TagV SH3b domain.

Figure S10. Superimposition of the putative TagV SH3b domain and Psm.

Table S1. Previously-described Type VI associated genes (Tags).

Table S2. Strains and plasmids used in this study.

Table S3. Primers used in this study.

References for Supplementary Information

```
TagV            ---------------MKTRITLTLLTALTLAGCSTPPPPPPALNNDALISSEVNGVTLQH    45
PAGR_g2450      ---------------MKKNLAFILLAGLLLSGCKTPP---PAVNDDTLVTSDVNGVKLVH    42
EBc_18420       --------MKCMRTNVKISIAFTLLAVLSLSGCKAPP---PPITDDTIVTSEVDGVKFTH    49
PAGR_g1655      -------MELNMKKMVNFRIALSLMFVFAVAGCKTP----PKMTDDTMVSSTVDGTTLIH    49
ES15_3853       ---------------MNMRIVLALLFVLGVAGCKAPP---PAVTDDTIVTSVVNGVTLTH    42
EAM_0571        --------------MVKIRIALSLLLVMSVVGCKTPP---PQITDDTVVSSTVDGVKLSY    43
EBc_05760       MSYAFHSKKNFMRIMVKIRIALSLLFVLTVAGCKAPP---PAMTDDTIISSTVDGVKLTY    57
                               ::  :.: *:  : : **.:*    * :.:*::::* *:*..: :

TagV            RAAVSAPKQFKPIGEEYRSLYAASIMSSPNYTGTAVGSLDNAAAFYALGEVENNWLAISA    105
PAGR_g2450      RHAVAAPTTFTPINQTWRALYKASVMTQPDYGGKVVRYLEAGKSFEVLGSVENHWLAIAD    102
EBc_18420       RHAVQAPTSFTPVNETYRALYNASVMTSPDFSGKNVRYLENGKPFTVLGEVENHWLAIAD    109
PAGR_g1655      RHAVAAPAQFSALNESYRALYPASVMTRPDFGGKVVETLKTGETYTVVGQVENNWLALGE    109
ES15_3853       RHAVTPPKEFEPVNEEYRAMYPASVMTRPDFGGKVVRQLETGKTYNVLGQVEGHWLAIAD    102
EAM_0571        RHAITPPQSFTPVNEEYRALYAASVMSRPDFGGKLVRHLDNGQTFTVLGSVENNWFAIAD    103
EBc_05760       RHAVQPPTSFTPVNEEYRALYDASVMNRPDFGGKLVSNLENGKPYTVLGSVENNWFAIAE    117
                * *:  *  *  :.: :*::* **:*. *:: *. *  *. .  : .:*.**.:*:*:.

TagV            IRG---------------------------GDLMGYIQANAGVPEARYKSTLRKDLPRRAR    139
PAGR_g2450      KPD---------------------------GMLTGYVPLKAGVERHRYDDTLRNDRPRPRK    136
EBc_18420       PDQ---------------------------EQLIGYVPFKAGVKTDLYDATIKSDRPRPRK    143
PAGR_g1655      EAQAEAPPAASDAKATDSKAAPAQATTSAIKVVGYVPFRAVVKSDLYDQTVKADQPKRRA    169
ES15_3853       QGE---------------------------EQLIGYVPLRAAVKSSLYDATVSKEIPK--L    134
EAM_0571        AGQ---------------------------EQLIGYVPLRAGVKSDLYNQTLKADQRRKRV    137
EBc_05760       QGQ---------------------------EQLIGYVPLRAVVKSDLYDKTVKADSRRKRV    151
                                               : **:  .* *    *. *:  :  :

TagV            ---AAKQDCVKVGGDSKACKNAGSATWILQ 166
PAGR_g2450      ---T-KQVCVDVGGDSKACRNTATATWILN 162
EBc_18420       ---NAKKVCVDVGGQSKACRNNATATWILE 170
PAGR_g1655      RAAAKKKTCVSVDGNSQACQNKNNGTWIIN 199
ES15_3853       RRKTAKANCVTVDGNSKACKNTKSGTWVID 164
EAM_0571        RAPAKKKTCVAVDGDSKACQNNNNGTWIID 167
EBc_05760       RAASK-KTCV-AAGDSKACQNSNSGTWIID 179
                        ** . *:*:**:*  ..**:::
```

Figure S1. Multiple sequence alignment of TagV and its homologues. Sequence titles for TagV homologues refer to their genomic identifiers as presented in Figure 1.

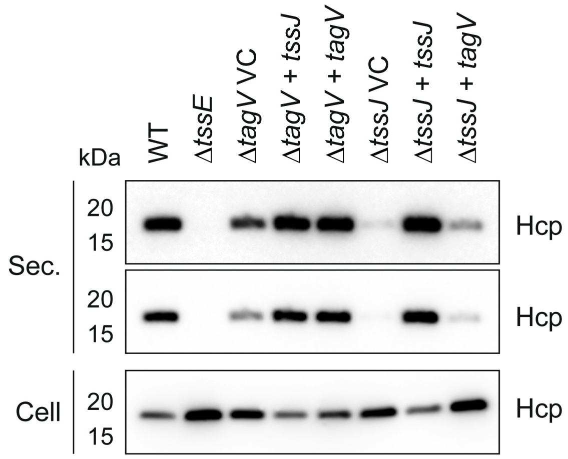

Figure S2. The reduced Hcp secretion observed in the Δ*tagV* and Δ*tssJ* mutants can be complemented by expression of the respective gene *in trans*. Levels of Hcp in the total cellular (Cell) and secreted (Sec.) protein fractions from wild type (WT) and mutant strains of *S. marcescens* carrying the vector control plasmid (pSUPROM, VC) or plasmids directing the expression of *tagV* (+ *tagV*) or *tssJ* (+ *tssJ*) as detected by immunoblot. Two different images of the same blot are shown for the secreted fractions to aid clarity of all bands.

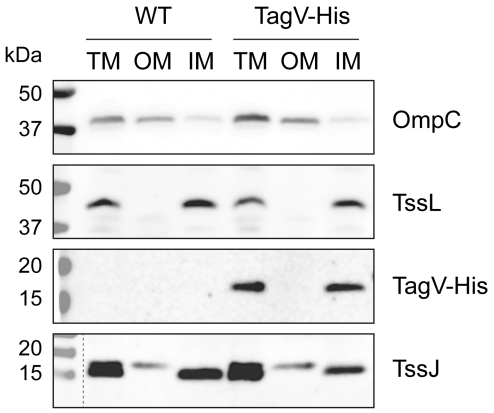

Figure S3. The outer membrane lipoproteins TagV and TssJ solubilise with the inner membrane of *S. marcescens* in selective detergent solubilisation experiments. Total membrane fractions (TM) of wild type (WT) and TagV-His were separated into outer membrane (OM) and inner membrane (IM) fractions by selective detergent (sarkosyl) solubilisation. Fractionated membranes were probed for the presence of OmpC (outer membrane control), TssL (inner membrane control), TagV-His or TssJ by immunoblot.

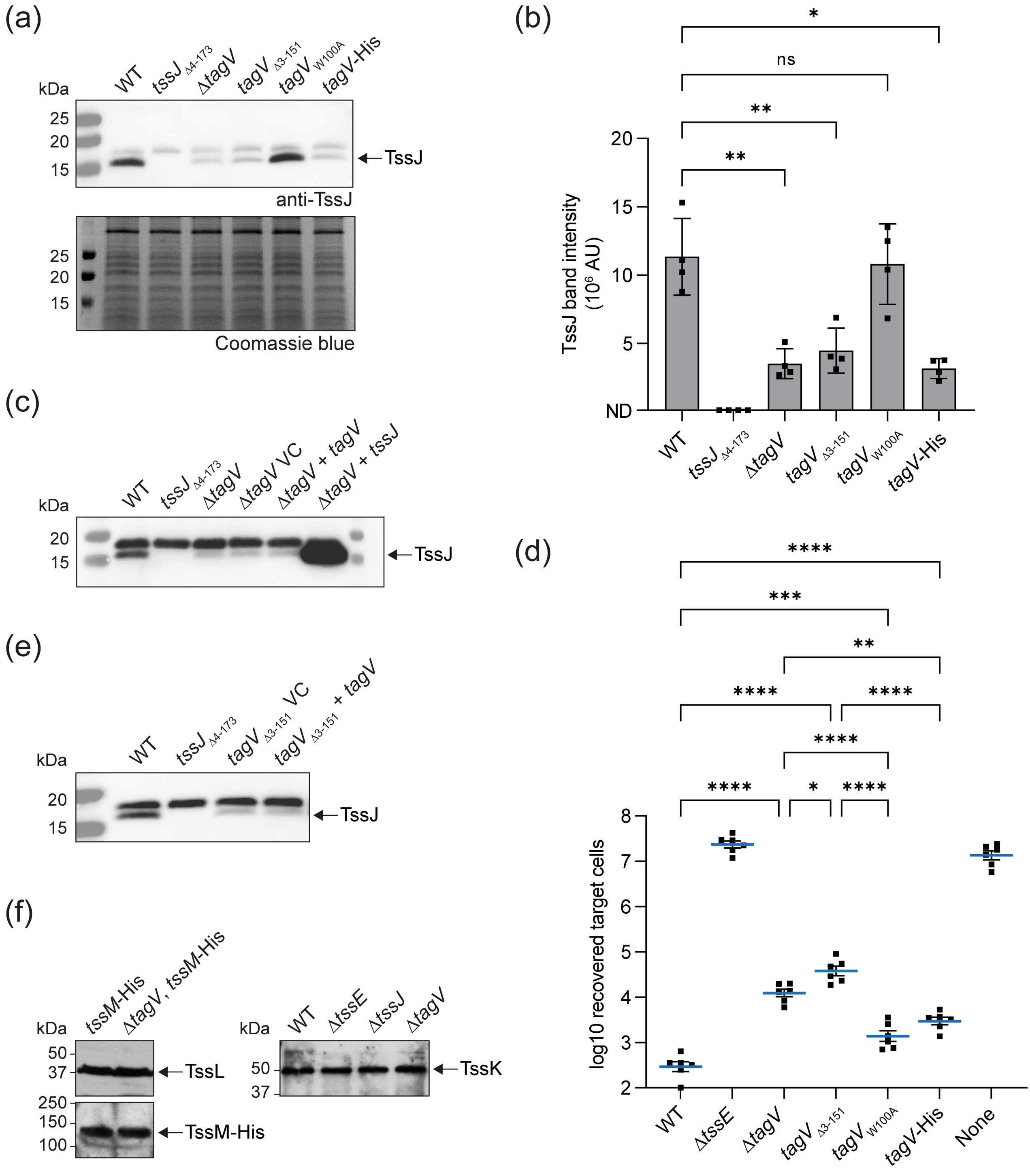

Figure S4. Genetic manipulation of *tagV* results in decreased TssJ production which cannot be restored by complementation with *tagV*. (a) Total cellular protein samples from wild type (WT) and mutant strains of *S. marcescens* were analysed for TssJ production by immunoblot. (b) TssJ band intensity as measured by densitometry. Data are displayed as mean +/- SEM from four independent experiments with individual data points overlaid. One-way ANOVA with Dunnett's multiple comparison test was performed (** P<0.01; * P<0.05; ns, not significant). (c) As in panel a except the Δ*tagV* mutant is carrying either the vector control plasmid (pSUPROM, VC) or plasmids directing the expression of *tagV* (+ *tagV*) or *tssJ* (+ *tssJ*) *in trans*. (d) Recovery of *P. fluorescens* KT02 target cells following co-culture with WT and mutant strains of *S. marcescens* at an initial ratio of 1:1 for four

hours. Data are displayed as mean +/- SEM (n=6) with individual data points overlaid. One-way ANOVA with Tukey's multiple comparison test was performed (**** $P<0.0001$; *** $P<0.001$; ** $P<0.01$; * $P<0.05$; for clarity, only selected comparisons are displayed). None, sterile media only. (e) As in panel c except the $tagV_{\Delta 3\text{-}151}$ mutant is carrying either the vector control plasmid (pSUPROM, VC) or plasmids directing the expression of *tagV* (+ *tagV*) *in trans*. (f) Total cellular protein samples from WT and mutant strains of *S. marcescens* were analysed for TssL, TssM and TssK production by immunoblot (TssM levels were detected by anti-His immunoblot of strains encoding TssM-His_6 at the normal chromosomal location, *tssM*-His).

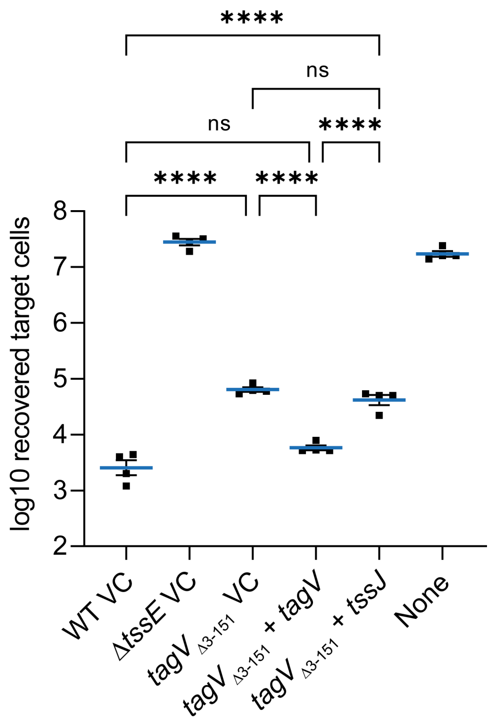

Figure S5. Expression of *tagV* but not *tssJ in trans* is able to restore T6SS activity in the *tagV*$_{\Delta 3\text{-}151}$ mutant. Recovery of *P. fluorescens* KT04 following co-culture with WT and mutant strains of *S. marcescens* carrying either the vector control plasmid (pSUPROM, VC) or plasmids directing the expression of *tagV* (+ *tagV*) or *tssJ* (+ *tssJ*) *in trans*. Data are displayed as mean +/- SEM (n=4) with individual data points overlaid. One-way ANOVA with Tukey's multiple comparison test was performed (**** $P<0.0001$; ns, not significant; for clarity, only selected comparisons are displayed). None, sterile media only.

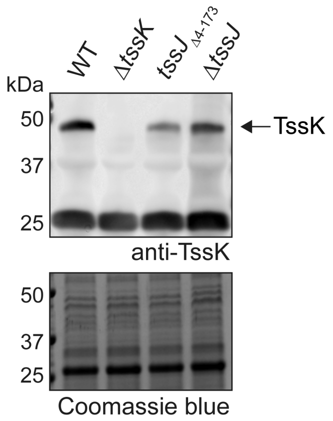

Figure S6. TssK production in strains carrying two different deletions in *tssJ*. Levels of TssK in total membrane protein fractions from wild type (WT) and mutant strains of *S. marcescens* were determined by immunoblot (*tssJ*$_{\Delta 4\text{-}173}$, in-frame deletion of amino acids 4-173 of 176; Δ*tssJ*, in-frame deletion of amino acids 4-28).

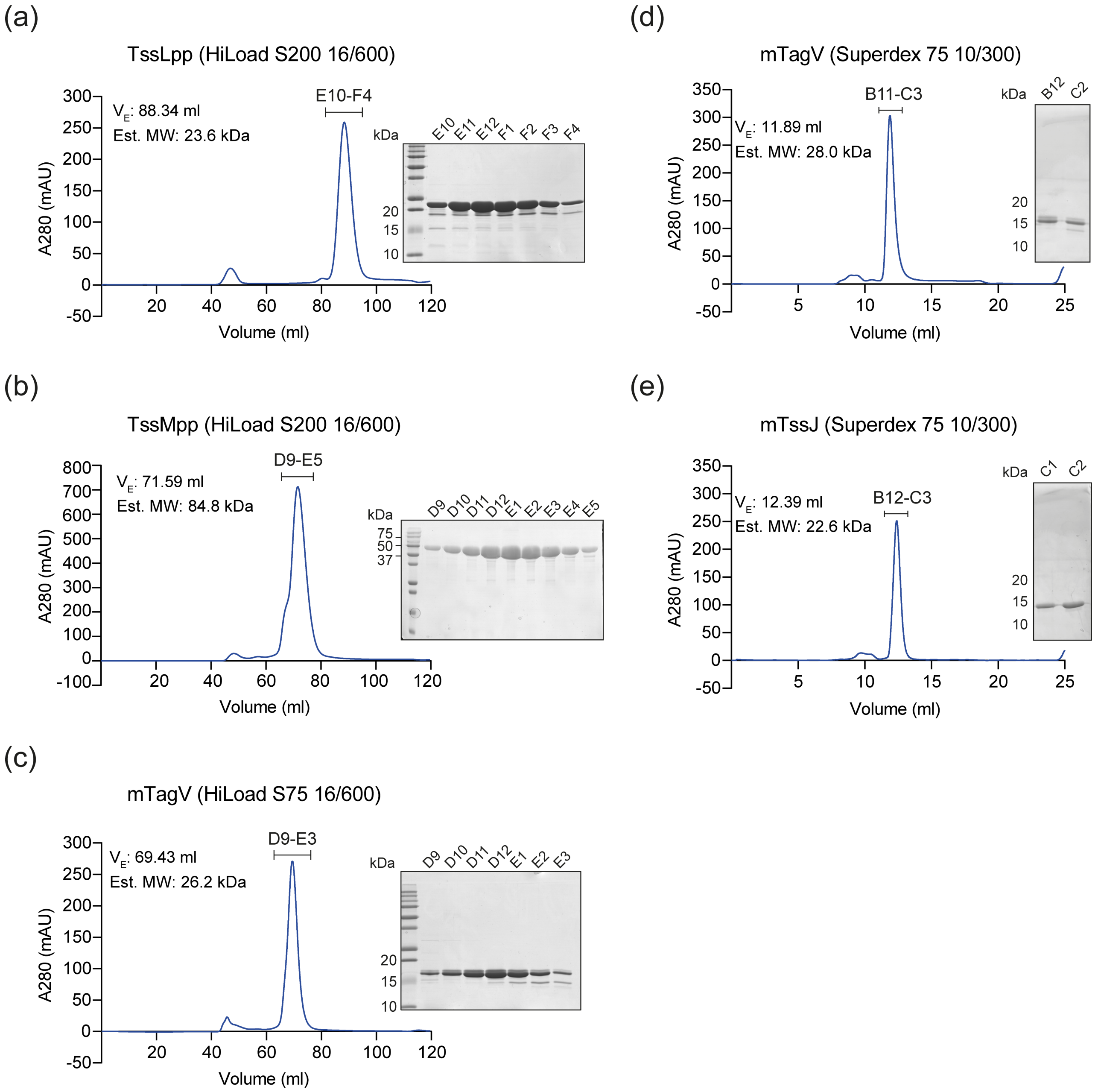

Figure S7. Integrity of proteins used for *in vitro* complex formation experiments. Affinity purified periplasmic domains of TssL (TssLpp, panel a) and TssM (TssMpp, panel b), and mature TagV (mTagV, panels c and d) and TssJ (mTssJ, panel e) proteins were separated by size exclusion chromatography using the column indicated. Protein fractions were visualised by SDS-PAGE and Coomassie staining. The elution volume (Ve) and estimated molecular weight (Est. Mw) of each peak, based on calibration of the column with protein standards, is noted in each case. Note that the data in panels d and e is the same as that used in Figure 4c for the individual mTagV and mTssJ proteins.

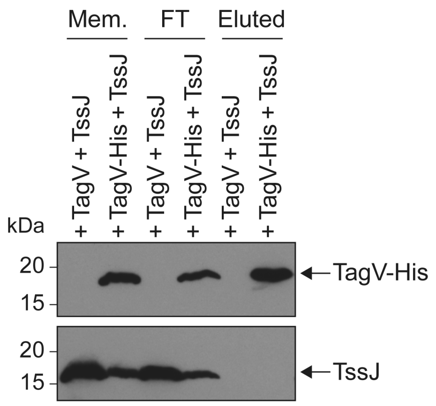

Figure S8. TssJ does not co-purify with TagV-His when both are expressed from a plasmid. Total membrane fractions prepared from *S. marcescens* Db10 Δ*tagV*Δ*tssJ* carrying plasmids directing the expression of TagV and TssJ (pSC2017) or TagV-His and TssJ (pSC2040) were incubated with Ni^{2+} beads and the membrane (input, Mem,), flowthrough (FT) and eluted fractions were probed for the presence of TagV-His and TssJ by immunoblot.

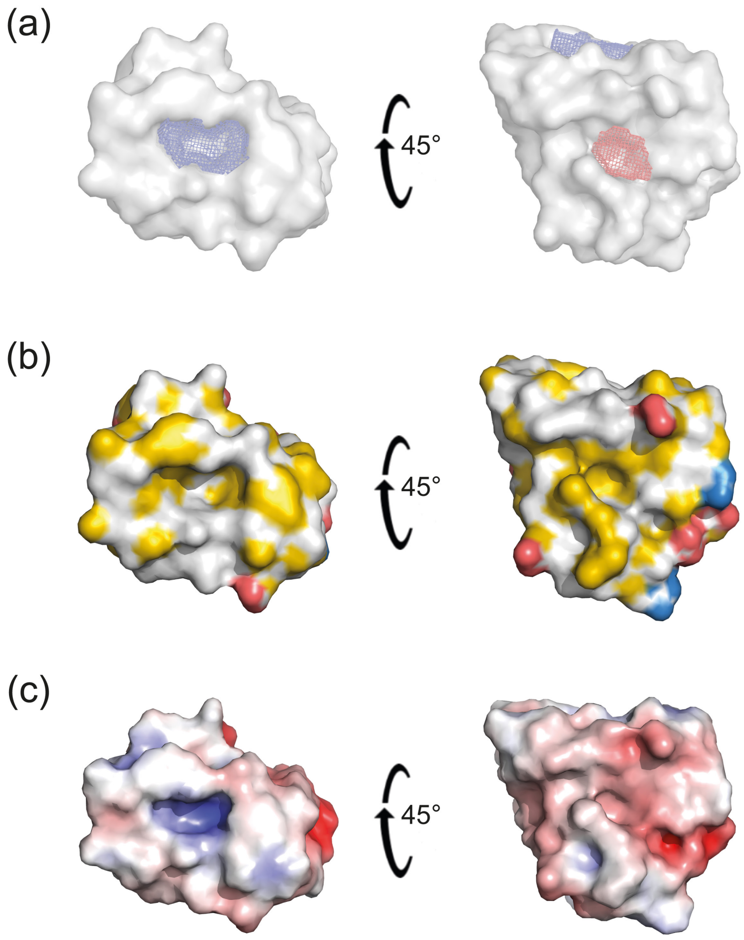

Figure S9. The predicted surface topology of the putative TagV SH3b domain. (a) Pockets P0 (blue mesh) and P1 (red mesh) identified by the DogSiteScorer algorithm (Volkamer *et al.*, 2012). (b) Surface hydrophobicity as determined using the YRB scheme proposed by Hagemans and colleagues (Hagemans *et al.*, 2015). Hydrophobic regions are highlighted in yellow, negatively charged groups of glutamate and aspartate are highlighted in red and positively charged functional groups of lysine and arginine are highlighted in blue. (c) Electrostatic potential as determined using the Adaptive Poisson-Boltzmann Solver for PyMol 2.0 (Baker *et al.*, 2001) and scaled from +5 kT/e (positive, blue) to −5 kT/e (negative, red).

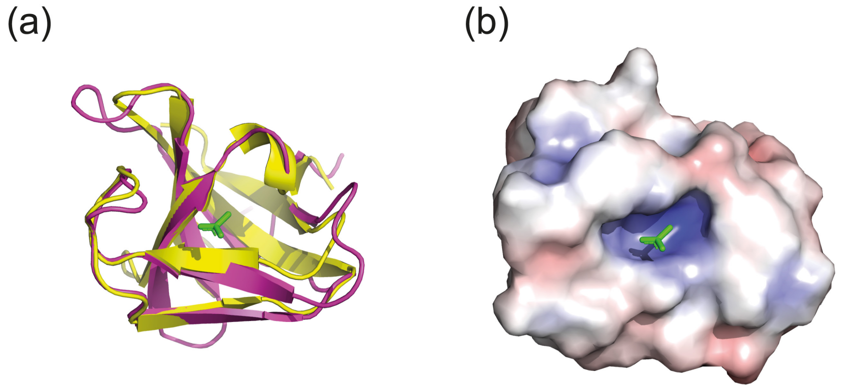

Figure S10. Superimposition of the putative TagV SH3b domain and Psm. (a) The putative TagV SH3b domain (yellow ribbon) is superimposed onto the structure of Psm (PDB 4krt, purple ribbon) in complex with an acetic acid molecule (green stick) with an RSMD of 0.719 Å. (b) The surface electrostatic potential of the superimposed TagV SH3b domain was determined using the Adaptive Poisson-Boltzmann Solver for PyMol 2.0 (Baker *et al.*, 2001) and is scaled from +5 kT/e (positive, blue) to −5 kT/e (negative, red).

Table S1. Previously-described Type VI associated genes (Tags).

Name	Description	Reference
TagA	DUF2169 domain-containing protein often encoded in T6SS gene clusters and originally named by Shalom and colleagues. Subsequently proposed to be an adaptor/chaperone protein for PAAR-containing effectors.	(Shalom *et al.*, 2007, Bondage *et al.*, 2016)
TagA / TsmA	ImpA_N domain-containing protein. Terminates sheath polymerisation and maintains 'long' TssA ($TssA_L$ or TsaC) in extended conformation.	(Santin *et al.*, 2018, Schneider *et al.*, 2019)
TagB	Pentapeptide repeat protein often encoded in T6SS gene clusters and originally named by Shalom and colleagues. Function unknown.	(Shalom *et al.*, 2007)
TagB / TsmB*	Sheath stabilisation in some T6SSs containing 'short' TssA ($TssA_S$ or TsaB). Also known as TagB1 and previously as TagX1. We propose it be named TsmB to fit with the nomenclature of Schneider *et al.*[7]	(Bernal *et al.*, 2021)
TagC	Protein of unknown function often encoded in T6SS gene clusters.	(Lennings *et al.*, 2018, Shalom *et al.*, 2007)
TagD	Class of PAAR protein, containing DUF4150 domain. Part of "PAAR_E" phylogenetic clade defined by Zhang and colleagues.	(Lennings *et al.*, 2018, Zhang *et al.*, 2021)
TagE	Usually known as PpkA. Thr protein kinase that phosphorylates Fha or TssL, activating T6SS assembly.	(Jiang *et al.*, 2019, Lin *et al.*, 2014, Fritsch *et al.*, 2013)
TagF	Negative post-translational regulator of T6SS assembly.	(Lin *et al.*, 2018, Silverman *et al.*, 2011)
TagG	Usually known as PppA. Thr protein phosphatase that dephosphorylates Fha, repressing T6SS assembly.	(Mougous *et al.*, 2007, Jiang *et al.*, 2019, Fritsch *et al.*, 2013)
TagH	Usually known as Fha. FHA domain-containing protein required for T6SS assembly in many T6SS. Can be substrate for PpkA phosphorylation.	(Mougous *et al.*, 2007, Jiang *et al.*, 2019, Ostrowski *et al.*, 2018)
TagI	Putative peptidoglycan-binding lipoprotein, function unknown.	(Shalom *et al.*, 2007)
TagJ	Sheath stabilisation in some T6SSs containing 'short' TssA (also known as $TssA_S$ or TsaB).	(Bernal *et al.*, 2021, Forster *et al.*, 2014)
TagK	Function unknown.	(Shalom *et al.*, 2007)
TagL	Also known as SciZ. Peptidoglycan-binding inner membrane protein, anchors membrane complex to the cell wall in T6SSs with 'short' TssL.	(Santin *et al.*, 2019, Nguyen *et al.*, 2021)
TagM	Putative outer membrane lipoprotein with peptidoglycan-binding domain, function unknown.	(Spiewak *et al.*, 2019, Shalom *et al.*, 2007)
TagN	Putative peptidoglycan-binding protein, function unknown.	(Aschtgen *et al.*, 2010, Ringel *et al.*, 2017)
TagO	Also known as VasI, function unknown.	(Zhang *et al.*, 2020, Moriel *et al.*, 2021)
TagP	Putative peptidoglycan-binding homologue of TssM. Thought to bind TssL.	(Aschtgen *et al.*, 2010)
TagQ	Anchors TagR to the outer membrane.	(Casabona *et al.*, 2013)
TagR	Positive regulator of PpkA kinase activity and thus Fha phosphorylation.	(Hsu *et al.*, 2009, Casabona *et al.*, 2013)
TagS	Integral membrane protein forming a membrane associated complex with TagT which co-operates with TagQR to regulate Fha phosphorylation by PpkA. TagQRST detects incoming T6SS attacks.	(Casabona *et al.*, 2013, Basler *et al.*, 2013)

TagT	ATPase forming a membrane associated complex with TagS which co-operates with TagQR to regulate Fha phosphorylation by PpkA. TagQRST detects incoming T6SS attacks.	(Casabona *et al.*, 2013, Basler *et al.*, 2013)
TagU	Unknown, variably described as a lipoprotein in *P. fluorescens* Pf29Arp and *P. chlororaphis* O6 and as a truncated ORF in *P. fluorescens* MFE01.	(Marchi *et al.*, 2013, Gallique *et al.*, 2017)
TagV	Not defined to date.	
TagW	Putative peptidoglycan binding protein, function unknown.	(Aschtgen *et al.*, 2010)
TagX	Also known as AsaE, peptidoglycan hydrolase required for T6SS transport across cell wall.	(Weber *et al.*, 2016, Spiewak *et al.*, 2019)
TagY	Transmembrane domain containing protein, function unknown.	(Spiewak *et al.*, 2019)
TagZ	Also known as AsaB, putative cytoplasmic membrane protein, function unknown.	(Spiewak *et al.*, 2019)
AsaA	Also known as TslA. Interacts with TssM and directs T6SS assembly to cell-cell contact sites.	(Li *et al.*, 2019, Ringel *et al.*, 2017, Weber *et al.*, 2016, Lin *et al.*, 2022)
RtkS	Interacts with PpkA and regulates T6SS firing.	(Ostrowski *et al.*, 2018)
TasL	Mediates cell-cell attachment which facilitates T6SS intoxication.	(Speare *et al.*, 2022)

*Name proposed here

Table S2. Strains and plasmids used in this study.

Name	Details	Reference
**<u>Bacterial strains</u>**		
Serratia marcescens		
Db10	Wild type strain	(Flyg *et al.*, 1980)
SJC11	Db10 Δ*tssE* (*SMDB11_2271*)	(Murdoch *et al.*, 2011)
LM04	Db10 Δ*tagV*; in-frame deletion in *tagV* (*SMDB11_2251*) ΔT3-T162	This study
LM31	Db10 Δ*tssJ*; in-frame deletion in *tssJ* (*SMDB11_2252*) ΔT4-S28	This study
LM40	Db10 Δ*tagV* Δ*tssJ*; in-frame deletions *tagV* ΔT3-T162, *tssJ* ΔT4-S28	This study
LM37	Db10 ΔtssE Δ*tssJ*; in-frame deletions Δ*tssE*, *tssJ* ΔT4-S28	This study
LM70	Db10 $tagV_{\Delta 3-151}$; in-frame deletion in *tagV* ΔT3-D151	This study
SJC10	Db10 $tssJ_{\Delta 4-173}$; in-frame deletion in *tssJ* ΔT4-K173	(Murdoch *et al.*, 2011)
BH03	Db10 *tagV*-His; encodes C-terminally His_6-tagged version of TagV at the native chromosomal location	(Mariano *et al.*, 2018)
MR10	Db10 $tagV_{W100A}$	This study
GM80	Db10 *tssM*-His; encodes C-terminally His_6-tagged version of TssM (SMDB11_2255) at the native chromosomal location	(Mariano *et al.*, 2018)
LM45	Db10 Δ*tagV*, *tssM*-His	This study
GE04	Db10 Δ*tssK* (*SMDB11_2253*)	(English *et al.*, 2014)
Pseudomonas fluorescens		
KT02	Sm^R derivative of *P. fluorescens* 55	(Murdoch *et al.*, 2011)
KT04	Sm^R Kan^R derivative of *P. fluorescens* 55	(Murdoch *et al.*, 2011)
Escherichia coli		
CC118λ*pir*	Donor strain for pKNG101-derived allelic exchange plasmids	(Herrero *et al.*, 1990)
HH26 pNJ5000	Mobilising strain for conjugal transfer	(Grinter, 1983)
**<u>Plasmids</u>**		
**Mutagenesis**		
pKNG101	Suicide vector for allelic exchange (Sm^R, *sacBR, mobRK2, oriR6K*)	(Kaniga *et al.*, 1991)
pSC1921	pKNG101-based allelic exchange plasmid for deletion of *tagV* (T3–T162)	This study
pSC2031	pKNG101-based allelic exchange plasmid for deletion of *tssJ* (T4–S28)	This study
pSC3430	pKNG101-based allelic exchange plasmid to introduce $tagV_{W100A}$ mutation	This study
pSC2069	pKNG101-based marker exchange plasmid for deletion of *tagV* (T3–D151)	This study
pSC1989	pKNG101-based marker exchange plasmid for addition of His_6 tag to C-terminus of *tagV*	(Mariano *et al.*, 2018)
**Protein production**		
pET15b-TEV	Protein overexpression vector for fusion with N-terminal His_6 tag under the control of T7 promoter. Derived from pET15b (Amp^R)	(Rao *et al.*, 2011)
pSC102	Protein overexpression vector for fusion with N-terminal His_6 tag under the control of T5 promoter (Ap^R, Kn^R).	(English *et al.*, 2014)
pSC534	TssLpp (G218-K406) cloned in pSC102	This study
pSC055	TssMpp (L527-P1211) cloned in pET15b-TEV	This study

pSC1952	mTagV (S19–Q166) cloned in pET15b-TEV	This study
pET15b-TEV_ SMA2252T	mTssJ (A30-D176) cloned in pET15b-TEV	(Rao *et al.*, 2011)
**Expression *in trans* in *S. marcescens***		
pSUPROM	Vector for constitutive expression of cloned genes under the control of the *E. coli tat* promoter (Kn^R)	(Murdoch *et al.*, 2011)
pSC066	Full length *tssJ* cloned in pSUPROM	(Murdoch *et al.*, 2011)
pSC1920	Full length *tagV* cloned in pSUPROM	This study
pSC2017	Full length *tagV* and *tssJ* cloned in pSUPROM	This study
pSC2040	Full length *tagV* with C-terminal His_6 tag and *tssJ* cloned in pSUPROM	This study
pSC1920 (W100A)	Full length *tagV* containing W100A point mutation cloned in pSUPROM	This study
pSC1920 (Q116E)	Full length *tagV* containing Q116E point mutation cloned in pSUPROM	This study
pSC1920 (Q116P)	Full length *tagV* containing Q116P point mutation cloned in pSUPROM	This study

Table S3. Primers used in this study.

Primer	Sequence (5’-3’)	Description
**TagV mutant construction**		
LM070	TGTGTCTAGATGCGCCAAACCGAGCACGTGCGCGC	Forward primer to clone upstream region of *tagV* into pKNG101 for Δ*tagV* production in Db10
LM071	TATAAAGCTTTTTCACAATGTCCTCTTTTTCTTGA	Reverse primer to clone upstream region of *tagV* into pKNG101 for Δ*tagV* production in Db10
LM072	TATAAAGCTTTGGATCCTCCAGTAGTCAGCACGCC	Forward primer to clone downstream region of *tagV* into pKNG101 for Δ*tagV* (T3–T162) production in Db10
LM073	TATAGGGCCCTACTTCGCTTCAGCGCAGCGGTAGG	Reverse primer to clone downstream region of *tagV* into pKNG101 for Δ*tagV* production in Db10
LM326	TATACTCGAGTTTCACAATGTCCTCTTTTTCTTGA	Reverse primer to clone upstream region of *tagV* into pKNG101 for Δ*tagV* production in Δ*tssJ*
LM327	TATACTCGAGTGGATCCTCCAGTAGTCAGCACGCC	Forward primer to clone downstream region of *tagV* into pKNG101 for Δ*tagV* (T3-T162) production in Δ*tssJ*
LM383	TATAAAGCTTAGCAAGGCGTGTAAAAACGC	Forward primer to clone downstream region of *tagV* into pKNG101 for Δ*tagV* (T3-D151) production in Db10
LM216	TATAAAGCTTCATCATCATCATCATCACTAGTCAGCA CGCCTGCGGGC	Forward primer to incorporate a C-terminal His_6 tag into *tagV*
MR009	ACAGGATCCAACGCGTTCAACATTCTGTTCGTC	Forward primer to clone upstream region of *tagV* into pKNG101 for $tagV_{W100A}$ production in Db10
MR004	GGAGATCGCCAGCGCGTTGTT	Reverse primer to clone upstream region of *tagV* into pKNG101 for $tagV_{W100A}$ production in Db10
MR005	AACAACGCGCTGGCGATCTCC	Forward primer to clone downstream region of *tagV* into pKNG101 for $tagV_{W100A}$ production in Db10
MR006	ACATCTAGAAGAACTGATCGCTGTCCAGCAG	Reverse primer to clone downstream region of *tagV* into pKNG101 for $tagV_{W100A}$ production in Db10
**TssJ mutant construction**		
LM214	TGTATCTAGACTCGGCATGCGCATCGTGCC	Forward primer to clone upstream region of *tssJ* into pKNG101 for Δ*tssJ* (T4-S28) production in Db10 and SJC11

SC2053	TATAAAGCTTCGTCATCATAGGAATCTCGTCG	Reverse primer to clone upstream region of *tssJ* into pKNG101 for Δ*tssJ* (T4-S28) production in Db10 and SJC11
LM310	TATAAAGCTTTCCGCCAAAAGCGTGCCGTC	Forward primer to clone downstream region of *tssJ* into pKNG101 for Δ*tssJ* (T4-S28) production in Db10 and SJC11
SC2469	TATAGGGCCCTATAGTTACTTCGCTTCAGCGC	Reverse primer to clone downstream region of tssJ into pKNG101 for Δ*tssJ* (T4-S28) production in Db10 and SJC11
**Protein expression**		
LM068	TATATCTAGAGTGAAAACACGGATTACCCTGACGC	Forward primer to clone *tagV* into pSUPROM
LM069	TATAAAGCTTCTACTGGAGGATCCAGGTGGCTGAG	Reverse primer to clone *tagV* into pSUPROM
LM293	TATAAAGCTTTCAGTCGACCTTTTTTACGG	Reverse primer to clone *tagV* - *tssJ* in pSUPROM
LM305	TATACTCGAGCTAGTGATGATGATGATGATG CTGGAGGATCCAGGTGGCTG	Reverse primer to clone *tagV* with C-term His_6 tag (with TssJ) in pSUPROM
LM306	TATACTCGAGTCAGCACGCCTGCGGGCGAC	Forward primer to clone *tssJ* (with *tagV*-His) in pSUPROM
SC2137	TATACATATGTTGGGCAACGGCGACATGTTC	Forward primer to clone *tssM*pp into pET15b-TEV
SC2120	TATAGGATCCCTACGGGCACGAGAAGGC	Reverse primer to clone *tssM*pp into pET15b-TEV
GE034	TATAGGATCCGGCGACAACACCAGCCC	Forward primer to clone *tssL*pp into pSC102
GE035	TATAGTCGACTTACTTTCCGGTTCCTTGCGG	Reverse primer to clone *tssL*pp into pSC102
**Site-directed mutagenesis**		
W100A_F	GGAAAACAACGCGCTGGCGATCTCCGCC	Site directed mutagenesis of pSC1920 to introduce W100A mutation
W100A_R	ACTTCGCCCAGCGCGTAG	Site directed mutagenesis of pSC1920 to introduce W100A mutation
Q116E_F	GGGGTATATCGAGGCCAACGCCG	Site directed mutagenesis of pSC1920 to introduce Q116E mutation
Q116E_R	ATCAGATCGCCCCCGCGG	Site directed mutagenesis of pSC1920 to introduce Q116E mutation
Q116P_F	GGGGTATATCCCGGCCAACGCCG	Site directed mutagenesis of pSC1920 to introduce Q116P mutation
Q116P_R	ATCAGATCGCCCCCGCGG	Site directed mutagenesis of pSC1920 to introduce Q116P mutation

References for Supplementary Information

Aschtgen, M.S., Thomas, M.S., and Cascales, E. (2010) Anchoring the type VI secretion system to the peptidoglycan: TssL, TagL, TagP... what else? *Virulence* **1**: 535-540.

Baker, N.A., Sept, D., Joseph, S., Holst, M.J., and McCammon, J.A. (2001) Electrostatics of nanosystems: application to microtubules and the ribosome. *Proc Natl Acad Sci U S A* **98**: 10037-10041.

Basler, M., Ho, B.T., and Mekalanos, J.J. (2013) Tit-for-tat: type VI secretion system counterattack during bacterial cell-cell interactions. *Cell* **152**: 884-894.

Bernal, P., Furniss, R.C.D., Fecht, S., Leung, R.C.Y., Spiga, L., Mavridou, D.A.I., and Filloux, A. (2021) A novel stabilization mechanism for the type VI secretion system sheath. *Proc Natl Acad Sci U S A* **118**.

Bondage, D.D., Lin, J.S., Ma, L.S., Kuo, C.H., and Lai, E.M. (2016) VgrG C terminus confers the type VI effector transport specificity and is required for binding with PAAR and adaptor-effector complex. *Proc Natl Acad Sci U S A* **113**: E3931-3940.

Casabona, M.G., Silverman, J.M., Sall, K.M., Boyer, F., Coute, Y., Poirel, J., Grunwald, D., Mougous, J.D., Elsen, S., and Attree, I. (2013) An ABC transporter and an outer membrane lipoprotein participate in posttranslational activation of type VI secretion in *Pseudomonas aeruginosa*. *Environ Microbiol* **15**: 471-486.

English, G., Byron, O., Cianfanelli, F.R., Prescott, A.R., and Coulthurst, S.J. (2014) Biochemical analysis of TssK, a core component of the bacterial Type VI secretion system, reveals distinct oligomeric states of TssK and identifies a TssK-TssFG subcomplex. *Biochem J* **461**: 291-304.

Flyg, C., Kenne, K., and Boman, H.G. (1980) Insect pathogenic properties of *Serratia marcescens*: phage-resistant mutants with a decreased resistance to Cecropia immunity and a decreased virulence to Drosophila. *J Gen Microbiol* **120**: 173-181.

Forster, A., Planamente, S., Manoli, E., Lossi, N.S., Freemont, P.S., and Filloux, A. (2014) Coevolution of the ATPase ClpV, the sheath proteins TssB and TssC, and the accessory protein TagJ/HsiE1 distinguishes type VI secretion classes. *J Biol Chem* **289**: 33032-33043.

Fritsch, M.J., Trunk, K., Diniz, J.A., Guo, M., Trost, M., and Coulthurst, S.J. (2013) Proteomic identification of novel secreted antibacterial toxins of the *Serratia marcescens* type VI secretion system. *Mol Cell Proteomics* **12**: 2735-2749.

Gallique, M., Decoin, V., Barbey, C., Rosay, T., Feuilloley, M.G., Orange, N., and Merieau, A. (2017) Contribution of the *Pseudomonas fluorescens* MFE01 Type VI Secretion System to Biofilm Formation. *PLoS One* **12**: e0170770.

Grinter, N.J. (1983) A broad-host-range cloning vector transposable to various replicons. *Gene* **21**: 133-143.

Hagemans, D., van Belzen, I.A., Moran Luengo, T., and Rudiger, S.G. (2015) A script to highlight hydrophobicity and charge on protein surfaces. *Front Mol Biosci* **2**: 56.

Herrero, M., de Lorenzo, V., and Timmis, K.N. (1990) Transposon vectors containing non-antibiotic resistance selection markers for cloning and stable chromosomal insertion of foreign genes in gram-negative bacteria. *J Bacteriol* **172**: 6557-6567.

Hsu, F., Schwarz, S., and Mougous, J.D. (2009) TagR promotes PpkA-catalysed type VI secretion activation in *Pseudomonas aeruginosa*. *Mol Microbiol* **72**: 1111-1125.

Jiang, X., Beust, A., Sappa, P.K., Volker, U., Dinse, T., Herglotz, J., and Reinhold-Hurek, B. (2019) Two Functionally Deviating Type 6 Secretion Systems Occur in the Nitrogen-Fixing Endophyte *Azoarcus olearius* BH72. *Front Microbiol* **10**: 459.

Kaniga, K., Delor, I., and Cornelis, G.R. (1991) A wide-host-range suicide vector for improving reverse genetics in gram-negative bacteria: inactivation of the *blaA* gene of *Yersinia enterocolitica*. *Gene* **109**: 137-141.

Lennings, J., West, T.E., and Schwarz, S. (2018) The *Burkholderia* Type VI Secretion System 5: Composition, Regulation and Role in Virulence. *Front Microbiol* **9**: 3339.

Li, L., Wang, Y.N., Jia, H.B., Wang, P., Dong, J.F., Deng, J., Lu, F.M., and Zou, Q.H. (2019) The type VI secretion system protein AsaA in *Acinetobacter baumannii* is a periplasmic protein physically interacting with TssM and required for T6SS assembly. *Sci Rep* **9**: 9438.

Lin, J.S., Pissaridou, P., Wu, H.H., Tsai, M.D., Filloux, A., and Lai, E.M. (2018) TagF-mediated repression of bacterial type VI secretion systems involves a direct interaction with the cytoplasmic protein Fha. *J Biol Chem* **293**: 8829-8842.

Lin, J.S., Wu, H.H., Hsu, P.H., Ma, L.S., Pang, Y.Y., Tsai, M.D., and Lai, E.M. (2014) Fha interaction with phosphothreonine of TssL activates type VI secretion in *Agrobacterium tumefaciens*. *PLoS Pathog* **10**: e1003991.

Lin, L., Capozzoli, R., Ferrand, A., Plum, M., Vettiger, A., and Basler, M. (2022) Subcellular localization of Type VI secretion system assembly in response to cell-cell contact. *EMBO J*: e108595.

Marchi, M., Boutin, M., Gazengel, K., Rispe, C., Gauthier, J.P., Guillerm-Erckelboudt, A.Y., Lebreton, L., Barret, M., Daval, S., and Sarniguet, A. (2013) Genomic analysis of the biocontrol strain *Pseudomonas fluorescens* Pf29Arp with evidence of T3SS and T6SS gene expression on plant roots. *Environ Microbiol Rep* **5**: 393-403.

Mariano, G., Monlezun, L., and Coulthurst, S.J. (2018) Dual Role for DsbA in Attacking and Targeted Bacterial Cells during Type VI Secretion System-Mediated Competition. *Cell Rep* **22**: 774-785.

Moriel, B., de Campos Prediger, K., de Souza, E.M., Pedrosa, F.O., Fadel-Picheth, C.M.T., and Cruz, L.M. (2021) In silico comparative analysis of *Aeromonas* Type VI Secretion System. *Braz J Microbiol* **52**: 229-243.

Mougous, J.D., Gifford, C.A., Ramsdell, T.L., and Mekalanos, J.J. (2007) Threonine phosphorylation post-translationally regulates protein secretion in *Pseudomonas aeruginosa*. *Nat Cell Biol* **9**: 797-803.

Murdoch, S.L., Trunk, K., English, G., Fritsch, M.J., Pourkarimi, E., and Coulthurst, S.J. (2011) The opportunistic pathogen *Serratia marcescens* utilizes type VI secretion to target bacterial competitors. *J Bacteriol* **193**: 6057-6069.

Nguyen, V.S., Spinelli, S., Cascales, E., Roussel, A., Cambillau, C., and Leone, P. (2021) Anchoring the T6SS to the cell wall: Crystal structure of the peptidoglycan binding domain of the TagL accessory protein. *PLoS One* **16**: e0254232.

Ostrowski, A., Cianfanelli, F.R., Porter, M., Mariano, G., Peltier, J., Wong, J.J., Swedlow, J.R., Trost, M., and Coulthurst, S.J. (2018) Killing with proficiency: Integrated post-translational regulation of an offensive Type VI secretion system. *PLoS Pathog* **14**: e1007230.

Rao, V.A., Shepherd, S.M., English, G., Coulthurst, S.J., and Hunter, W.N. (2011) The structure of *Serratia marcescens* Lip, a membrane-bound component of the type VI secretion system. *Acta Crystallogr D Biol Crystallogr* **67**: 1065-1072.

Ringel, P.D., Hu, D., and Basler, M. (2017) The Role of Type VI Secretion System Effectors in Target Cell Lysis and Subsequent Horizontal Gene Transfer. *Cell Rep* **21**: 3927-3940.

Santin, Y.G., Camy, C.E., Zoued, A., Doan, T., Aschtgen, M.S., and Cascales, E. (2019) Role and Recruitment of the TagL Peptidoglycan-Binding Protein during Type VI Secretion System Biogenesis. *J Bacteriol* **201**.

Santin, Y.G., Doan, T., Lebrun, R., Espinosa, L., Journet, L., and Cascales, E. (2018) In vivo TssA proximity labelling during type VI secretion biogenesis reveals TagA as a protein that stops and holds the sheath. *Nat Microbiol* **3**: 1304-1313.

Schneider, J.P., Nazarov, S., Adaixo, R., Liuzzo, M., Ringel, P.D., Stahlberg, H., and Basler, M. (2019) Diverse roles of TssA-like proteins in the assembly of bacterial type VI secretion systems. *EMBO J* **38**: e100825.

Shalom, G., Shaw, J.G., and Thomas, M.S. (2007) In vivo expression technology identifies a type VI secretion system locus in *Burkholderia pseudomallei* that is induced upon invasion of macrophages. *Microbiology* **153**: 2689-2699.

Silverman, J.M., Austin, L.S., Hsu, F., Hicks, K.G., Hood, R.D., and Mougous, J.D. (2011) Separate inputs modulate phosphorylation-dependent and -independent type VI secretion activation. *Mol Microbiol* **82**: 1277-1290.

Speare, L., Woo, M., Dunn, A.K., and Septer, A.N. (2022) A Putative Lipoprotein Mediates Cell-Cell Contact for Type VI Secretion System-Dependent Killing of Specific Competitors. *mBio* **13**: e0308521.

Spiewak, H.L., Shastri, S., Zhang, L., Schwager, S., Eberl, L., Vergunst, A.C., and Thomas, M.S. (2019) *Burkholderia cenocepacia* utilizes a type VI secretion system for bacterial competition. *MicrobiologyOpen*: e774.

Volkamer, A., Kuhn, D., Grombacher, T., Rippmann, F., and Rarey, M. (2012) Combining global and local measures for structure-based druggability predictions. *J Chem Inf Model* **52**: 360-372.

Weber, B.S., Hennon, S.W., Wright, M.S., Scott, N.E., de Berardinis, V., Foster, L.J., Ayala, J.A., Adams, M.D., and Feldman, M.F. (2016) Genetic Dissection of the Type VI Secretion System in *Acinetobacter* and Identification of a Novel Peptidoglycan Hydrolase, TagX, Required for Its Biogenesis. *MBio* **7**.

Zhang, J., Arif, M., Shen, H., Hu, J., Sun, D., Pu, X., Yang, Q., and Lin, B. (2020) Genomic divergence between *Dickeya zeae* strain EC2 isolated from rice and previously identified strains, suggests a different rice foot rot strain. *PLOS ONE* **15**: e0240908.

Zhang, Z., Liu, Y., Zhang, P., Wang, J., Li, D., and Li, Y.Z. (2021) PAAR Proteins Are Versatile Clips That Enrich the Antimicrobial Weapon Arsenals of Prokaryotes. *mSystems* **6**: e0095321.
